# An integrative pan-cancer-wide analysis of epigenetic enzymes reveals universal patterns of epigenomic deregulation in cancer

**DOI:** 10.1186/s13059-015-0699-9

**Published:** 2015-07-14

**Authors:** Zhen Yang, Allison Jones, Martin Widschwendter, Andrew E. Teschendorff

**Affiliations:** Key Laboratory of Computational Biology, CAS-MPG Partner Institute for Computational Biology, 320 Yue Yang Road, Shanghai, 200031 China; Department of Women’s Cancer, University College London, 74 Huntley Street, London, WC1E 6AU UK; Statistical Cancer Genomics, Paul O’Gorman Building, UCL Cancer Institute, University College London, 72 Huntley Street, London, WC1E 6BT UK

## Abstract

**Background:**

One of the most important recent findings in cancer genomics is the identification of novel driver mutations which often target genes that regulate genome-wide chromatin and DNA methylation marks. Little is known, however, as to whether these genes exhibit patterns of epigenomic deregulation that transcend cancer types.

**Results:**

Here we conduct an integrative pan-cancer-wide analysis of matched RNA-Seq and DNA methylation data across ten different cancer types. We identify seven tumor suppressor and eleven oncogenic epigenetic enzymes which display patterns of deregulation and association with genome-wide cancer DNA methylation patterns, which are largely independent of cancer type. In doing so, we provide evidence that genome-wide cancer hyper- and hypo- DNA methylation patterns are independent processes, controlled by distinct sets of epigenetic enzyme genes. Using causal network modeling, we predict a number of candidate drivers of cancer DNA hypermethylation and hypomethylation. Finally, we show that the genomic loci whose DNA methylation levels associate most strongly with expression of these putative drivers are highly consistent across cancer types.

**Conclusions:**

This study demonstrates that there exist universal patterns of epigenomic deregulation that transcend cancer types, and that intra-tumor levels of genome-wide DNA hypomethylation and hypermethylation are controlled by distinct processes.

**Electronic supplementary material:**

The online version of this article (doi:10.1186/s13059-015-0699-9) contains supplementary material, which is available to authorized users.

## Background

Epigenetic alterations are by now a well-recognized cancer hallmark [1]. One of the most intriguing and exciting observations to have recently emerged in cancer genomics is the subtle interplay between genetic and epigenetic mutations [2, 3]. Specifically, many epigenetic enzymes (EEs), including chromatin modifiers, have been found to exhibit genetic mutations in cancer, often resulting in a genome-wide deregulation of the DNA methylation (DNAm) landscape [2–6]. For example, alteration of genome-wide DNAm and gene expression patterns has been observed in acute monocytic leukemias carrying *DNMT3A* mutations [7]. Another example is mutation of *IDH1* (isocitrate dehydrogenase 1), which establishes a hypermethylator phenotype in glioma [8, 9]. Mutations in the DNA demethylation pathway, affecting genes such as *TET1*, *TET2* and *TET3*, have been discovered in myeloproliferative neoplasms and acute myeloid leukemia [10]. In general, it is thought that deregulation of global chromatin, DNAm and gene expression patterns may help cancer cells evolve more swiftly, fueling intra-tumor heterogeneity and resulting in increased invasiveness, drug resistance and metastatic potential [11–15]. Indeed, several of the mutations targeting EE genes are considered to be key driver mutations in specific cancers, while a few are also targetable with existing and upcoming therapies [1–3, 16, 17].

Given the emerging role of EE genes as drivers of the carcinogenic process, it has become of paramount interest to study their patterns of deregulation in cancer [18, 19]. Specifically, here we aimed to determine if EE genes exhibit consistent patterns of deregulation across different cancer types and, if so, whether these patterns dictate corresponding changes in the cancer DNA methylome [20]. In order to assess the patterns of deregulation of EE genes in cancer, we decided to anchor our analysis on their mRNA expression patterns. There are three reasons for focusing on gene expression. First, the frequency of genetic mutation of any given EE in any given cancer type is usually quite low. Second, the effect of an observed mutation on gene function can be hard to predict. Thus, although mRNA expression may not always correlate with gene activity, the same is true for specific mutations. Third, and most importantly, functional disruption of an EE may also be caused by a mechanism other than mutation, for instance by amplification or deletion, or by DNAm itself [21, 22].

Therefore, we posited that by analyzing matched RNA-Seq and DNAm data in a pan-cancer-wide study of The Cancer Genome Atlas (TCGA) data, that we would be able to identify universal patterns of covariation between expression and DNAm, determined by specific EE genes, thus allowing us to pinpoint the most important regulators of the cancer epigenome. To this end, we here conduct a pan-cancer-wide integrative analysis focusing on a large class of EE genes, including all main writers, readers, editors and erasers of the epigenome.

## Results

### Pan-cancer-wide differential expression analysis of EE genes

**Fig. 1:**
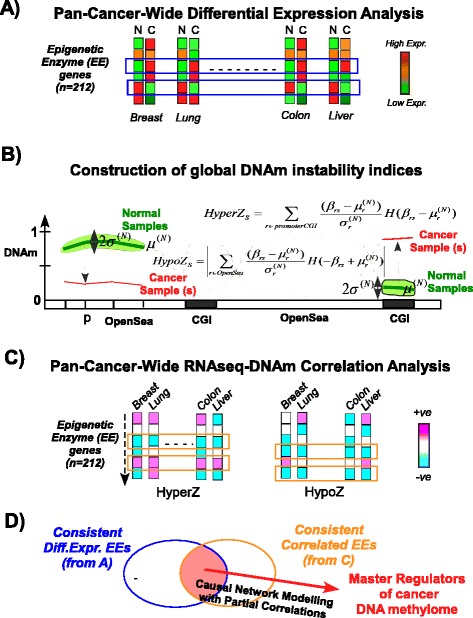
Identification of master epigenetic regulators of the cancer DNA methylome. **a** First, we conduct a pan-cancer-wide (TCGA) differential expression analysis of a comprehensive list of 212 “epigenetic enzyme” (*EE*) genes, defined as genes which play a role in modifying or regulating epigenetic marks, in order to identify those which exhibit consistent up- or downregulation across different cancer types. *N* normal, *C* cancer. **b** Since EE genes may control the epigenome, including the DNA methylome, we computed for each cancer sample two epigenetic instability indices (*HyperZ* and *HypoZ*), reflecting the global deviation in DNAm patterns from a normal reference (obtained using the corresponding normal tissue specimens). Briefly, the HyperZ index measures aberrant hypermethylation over promoter CpG islands (*CGI*) in a given cancer sample, whereas HypoZ measures aberrant hypomethylation over opensea probes (intergenic regions of low CpG density). **c** Third, we use the matched RNA-Seq and DNAm data of TCGA tumor samples to conduct a pan-cancer-wide correlation analysis between the expression levels of EE genes and these two epigenetic instability indices in order to identify EE genes whose expression variation associates with aberrant cancer DNAm. **d** Finally, we use causal network modeling of the EE genes which show consistent differential expression and correlation with HyperZ/HypoZ across cancer types to identify the subset of EE genes which appear to control the global variations in DNAm (HyperZ/HypoZ). The causal modeling uses partial correlations to eliminate (indirect) associations between EE gene expression and HyperZ/HypoZ which are mediated by DNAm changes driven by other EE genes

**Fig. 2:**
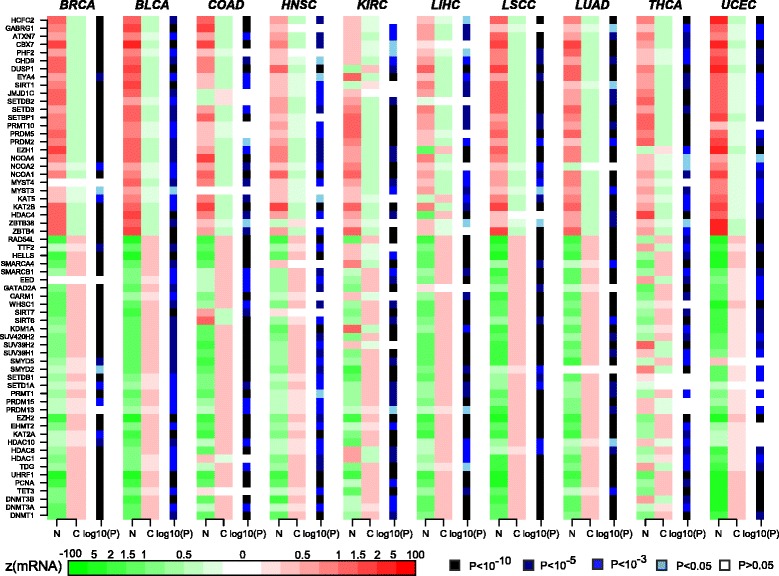
Pan-cancer-wide differential expression analysis of epigenetic enzyme genes. Heatmaps of average expression in normal (*N*) and cancer (*C*) tissue, across ten different TCGA cancer types (*BRCA* breast cancer, *BLCA* bladder cancer, *COAD* colon adenomacarcinoma, *HNSC* head and neck squamous carcinoma, *KIRC* kidney renal carcinoma, *LIHC* liver hepatocellular carcinoma, *LSCC* lung squamous cell carcinoma, *LUAD* lung adenomacarcinoma, *THCA* thyroid cancer, *UCEC* uterine cervix endometrial carcinoma) for 62 EE genes which showed consistent differential expression in at least eight of the ten tissue types. The significance level of differential expression is indicated by the sidebars for each heatmap

Given the emerging importance of EE genes, we compiled a comprehensive list of such genes from several recent reviews and a literature search [2, 3]. The list contained a total of 212 genes, including writers, readers, editors and erasers of the most important epigenetic marks, including histone and DNAm marks (Table S1 in Additional file 1). Although previous studies have already shown that a subset of these EE genes exhibit differential expression in cancer (e.g., [18]), we wanted to re-assess this using a more comprehensive list of EE genes and also using fresh high-quality RNA-Seq data from TCGA consortium. Gene-normalized RNA-Seq data for ten cancer types from TCGA [23–32] were downloaded and subjected to a quality control procedure designed to assess the relative amount of variation associated with biological versus technical factors (i.e., batch effects; Supplemental Methods, Figure S1, and Table S2 in Additional file 1). Differentially expressed EE genes were identified across all cancer types (Additional file 2). Kidney, breast and thyroid cancer exhibited the highest fraction (~77 %) of differentially expressed EE genes (Benjamini Hochberg false discovery rate <0.05), whilst bladder and endometrial cancer exhibited the lowest fractions (~53 %) (Additional file 2). EE genes consistently over- or under-expressed across all or most cancer types are of particular interest, since these may represent an initial candidate list of epigenetic oncogenes or tumor suppressors (Fig. 1a). We identified a total of 62 EE genes which were consistently deregulated across at least eight of the ten cancer types, with 35 of these upregulated and representing putative oncogenes, and with the remaining 27 exhibiting downregulation (Fig. 2).

To assess the overall statistical significance of these numbers, we estimated the corresponding counts for a random set of 212 genes using an analytic binomial model (“Materials and methods”; Table S3 in Additional file 1). Specifically, we estimated that for a random choice of 212 genes, we would only expect about 0.54 ± 0.74, i.e., one or no genes, to be significantly upregulated in at least eight of the ten cancer types, and only about 0.89 ± 0.94, i.e., none to two genes, to be significantly downregulated in at least eight datasets (“Materials and methods”; Table S4 in Additional file 1). From this, we estimated the null probabilities of observing 35 upregulated and 27 downregulated EE genes in as many as eight of the ten cancer types to be as low as *P* < 10^−50^ (in the case of upregulation) and *P* < 10^−30^ (in the case of downregulation) (binomial test *P* values; “Materials and methods”).

Among the upregulated genes were those encoding well-known enzymes such as *EZH2*, a histone methyltransferase catalyzing H3K27me3, and which has already been identified as a potential therapy target for cancer [33, 34]. Notably, the upregulated list also included genes encoding the methyl-transferase enzymes DNMT1, DNMT3A, and DNMT3B, various histone deacetylases (HDAC1, HDAC8, HDAC10), as well as PRMT1 (a histone methyltransferase), KDM1A (a histone demethylase) and SMARCB1 (a chromatin remodeling helicase). One of the genes with the most consistent and marked overexpression was *UHRF1*, which encodes a member of the RING-finger type E3 ubiquitin ligases which has been implicated in the maintenance of DNAm by interaction with DNA methyltransferases. Among the underexpressed genes, we observed the DNAm reader *ZBTB4*, whose expression has been significantly correlated with relapse-free survival [35], as well as the polycomb component and K36 reader, *CBX7*, which has already been implicated as a tumor suppressor [36–40]. Interestingly, this list of putative epigenetic tumor suppressors included several histone methyltransferases (*EZH1*, *SETD3*, *SETBP1*, *PRDM2* and *PRDM5*) and histone acetyltransferases (*NCOA1*, *NCOA2*, *NCOA4*, *KAT5*, *KAT2B*), but only one histone acetylation editor (*SIRT1*) and only one histone deacetylase (*HDAC4*).

### Genome-wide levels of DNA hypermethylation and hypomethylation are only weakly correlated in cancer

**Fig. 3:**
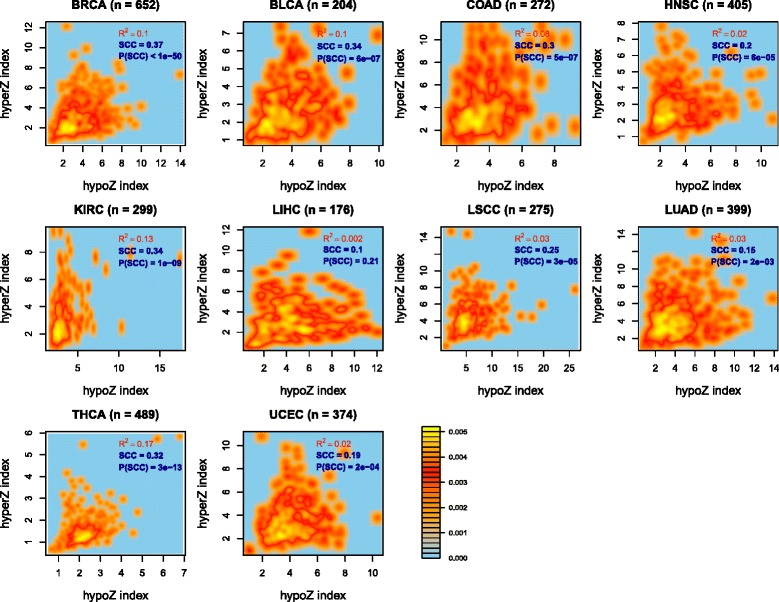
Genome-wide hypomethylation and hypermethylation correlate weakly. For each cancer type (*BRCA* breast cancer, *BLCA* bladder cancer, *COAD* colon adenomacarcinoma, *HNSC* head and neck squamous carcinoma, *KIRC* kidney renal carcinoma, *LIHC* liver hepatocellular carcinoma, *LSCC* lung squamous cell carcinoma, *LUAD* lung adenomacarcinoma, *THCA* thyroid cancer, *UCEC* uterine cervix endometrial carcinoma), we display two-dimensional density plots (*bright yellow* indicates highest density) illustrating the distribution of tumors in the plane defined by the HyperZ and HypoZ indices. The number of tumors is given above each panel. For each cancer type, we provide the Spearman correlation coefficient (*SCC*), its *P* value, as well as the R^2^ value for a linear regression

Given that many EE genes are aberrantly expressed in cancer and given their role in modulating/regulating the epigenome, including, potentially, the DNA methylome, we aimed to identify those genes which might control the aberrant DNAm patterns seen in cancer. To this end, we first needed to define a measure of aberrant DNAm in individual cancer samples. We adopted a strategy similar to that used by us previously [41], defining DNAm “instability” indices by comparison of a cancer’s DNAm profile with a normal reference obtained from the corresponding normal tissue samples of TCGA. Since DNA hyper- and hypomethylation may be controlled by distinct epigenetic pathways, we decided to construct two separate indices for each individual cancer sample, one measuring the “hypermethylation” deviation from the normal reference and another measuring the degree of “hypomethylation” (Fig. 1b). Since the baseline level of DNAm in a normal sample depends largely on CpG density, the two indices were constructed for distinct regions of the genome (“Materials and methods”). Briefly, for a given cancer sample, the index was constructed by averaging the Z scores (as computed relative to the average and variance of the normal samples) across all probe clusters falling within the appropriate genomic region: opensea probes for the “HypoZ” index, and promoter CGIs for the “HyperZ” index (“Materials and methods”, Fig. 1b). Thus, for each cancer sample *s*, we obtained an overall *HyperZ*_s_, and separately, a *HypoZ*_*s*_ index, reflecting the global level of aberrant DNA hypermethylation and hypomethylation in that sample, respectively. Plotting the HyperZ and HypoZ instability indices against each other for all cancer samples of a given tissue type revealed no strong correlation between them, although associations were significant owing to large sample sizes (Fig. 3). An even weaker correlation was observed if the indices were computed by restricting to loci with only significant Z scores (“Materials and methods”; Figure S2 in Additional file 1). The lack of a strong correlation between the two DNAm indices, across so many cancer types, is consistent with studies suggesting that cancer hyper- and hypomethylation constitute independent processes in tumor progression [41–43].

To demonstrate the biological significance of these DNAm instability indices, we asked whether they differ between different cancer subtypes. We performed this analysis for breast cancer, for which a number of transcriptomic “intrinsic subtypes” have been determined and firmly established [44, 45]. Both HyperZ and HypoZ indices were highest in the luminal B subtype (Figure S3 in Additional file 1). In fact, both indices differed between the luminal A and B subtypes, with a more widespread DNAm deregulation in the luminal B estrogen receptor-positive subtype, consistent with a previous report [32]. Interestingly, however, the HyperZ index was not only highest in luminal B tumors, but also in the HER2+ subtype, whereas the HypoZ index was significantly lower in HER2+ tumors compared with luminal B. Thus, this shows that luminal B breast cancers exhibit more widespread deregulation of DNAm patterns than HER2+ breast cancers.

### Pan-cancer-wide correlation analysis between EE gene expression and global DNAm reveals universal patterns of epigenomic deregulation

**Fig. 4:**
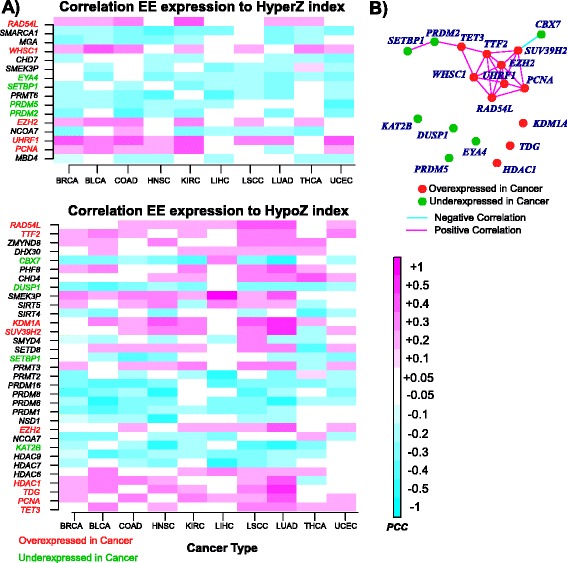
Pan-cancer-wide correlation analysis between EE expression and DNA methylation. **a** Heatmaps of Pearson correlation coefficients between mRNA expression of EE genes and the HyperZ or HypoZ indices, as assessed across cancers from ten different TCGA cancer types (*BRCA* breast cancer, *BLCA* bladder cancer, *COAD* colon adenomacarcinoma, *HNSC* head and neck squamous carcinoma, *KIRC* kidney renal carcinoma, *LIHC* liver hepatocellular carcinoma, *LSCC* lung squamous cell carcinoma, *LUAD* lung adenomacarcinoma, *THCA* thyroid cancer, *UCEC* uterine cervix endometrial carcinoma). Only EE genes exhibiting significant and directionally consistent correlations in at least six of the ten cancer types are shown. The subset of EE genes which also show significant and directionally consistent differential expression changes between normal and cancer in at least eight of the ten cancer types are colored, with *red* indicating overexpression in cancer, *green* underexpression. Those shown in *black* indicate that these were not consistently differentially expressed across the ten cancer types. **b** Correlation network meta-analysis (across all ten cancers) of the main epigenetic oncogenes and tumor suppressors which are associated with HyperZ or HypoZ

Having found that global aberrant DNA hypermethylation and hypomethylation are not generally correlated, we next decided to investigate if these two distinct DNAm instability indices are determined by the expression patterns of specific EE genes. Using matched RNA-Seq and DNAm data for cancers of a given tissue type, we computed Pearson correlations between the EE genes’ expression profiles and these epigenetic instability indices, separately for each tissue type (Fig. 1c). This revealed many significant associations between expression of EE genes and the HyperZ and HypoZ instability indices (“Materials and methods”; Figures S4 and S5 in Additional file 1). Although many of these associations and their directionality were cancer-specific, we also observed several associations which were consistent across cancer types (“Materials and methods”; Fig. 4a): a total of 16 genes exhibited significant correlations of consistent directionality (five positive and 11 negative correlations) with the HyperZ index, in at least six of the ten cancer types, whereas there were 33 genes which did so (18 positive and 15 negative correlations) with the HypoZ index (Fig. 4a). In order to assess the overall statistical significance of these numbers, we used an analytical binomial model to estimate the expected numbers for a randomly selected set of 212 genes (“Materials and methods”; Tables S5 and S6 in Additional file 1). In every case, the observed numbers of significantly and consistently correlated EE genes with HyperZ/HypoZ across at least six of the ten cancer types were significantly higher than those of a randomly selected set of 212 genes (“Materials and methods”; Table S6 in Additional file 1; binomial test *P* values ranged from 0.001 to 10^−24^). Thus, the consistent correlations across six of the ten cancer types as depicted in Fig. 4a are highly unlikely to be due to random chance. Generally speaking, EE genes exhibiting consistent correlations with the HyperZ index did not do so with the HypoZ index, although there were a few exceptions to this rule, which included *RAD54L*, *SMEK3P*, *SETBP1*, *NCOA7*, *EZH2* and *PCNA* (Fig. 4a).

### Candidate epigenetic regulators of cancer DNAm

To identify EE genes which may represent master regulators of the global DNAm patterns in cancer, we focused on those EE genes which exhibited consistent differential expression and DNAm instability correlation patterns across cancer types (Fig. 1d). We identified a total of 18 such genes, with seven of them exhibiting underexpression in cancer (*EYA4*, *SETBP1*, *PRDM2*, *PRDM5*, *CBX7*, *DUSP1* and *KAT2B*), and 11 exhibiting overexpression (*RAD54L*, *WHSC1*, *EZH2*, *UHRF1*, *PCNA*, *TTF2*, *KDM1A*, *SUV39H2*, *HDAC1*, *TDG* and *TET3*) (Fig. 4a). Remarkably, the 11 cancer overexpressed EEs always exhibited positive correlations with HyperZ and/or HypoZ, whereas the seven underexpressed genes always exhibited anticorrelations (Fig. 4a), clearly indicating that in both cases it is the deregulation of gene expression from the normal reference which associates with widespread changes in DNAm.

Next, we asked if the associations of these 18 genes are independent of each other, or, if instead, they are highly correlated. Computing correlations in mRNA expression between these 18 genes across all cancer samples of a given type, and combining the results in a meta-analysis over cancer types (“Materials and methods”), revealed a core cluster of positively correlated oncogenes, which included *UHRF1*, *EZH2*, *TTF2*, *SUV39H2*, *PCNA*, *WHSC1*, and *RAD54L* (Fig. 4b). This analysis also revealed a number of epigenetic oncogenes (*KDM1A*, *HDAC1*, *TDG*) and tumor suppressors (*KAT2B*, *PRDM5*, *DUSP1*, *EYA4*) which did not correlate significantly with any of the others, suggesting that these may affect cancer DNAm patterns independently of each other (Fig. 4b).

**Fig. 5:**
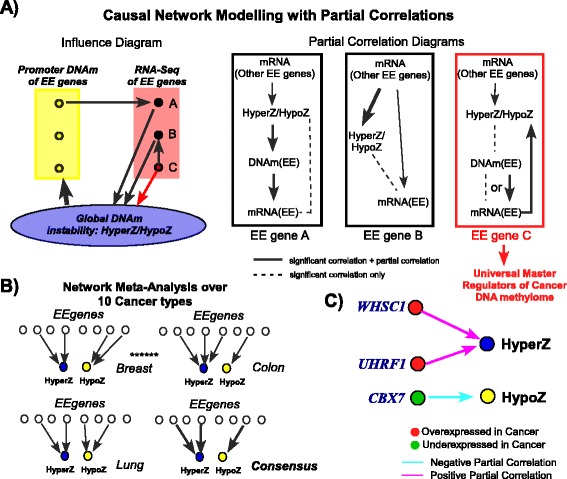
Causal network modeling meta-analysis. **a** Influence diagram depicts how correlations between expression of EE genes and HyperZ/HypoZ could arise. For gene A, global changes in DNAm affect the DNAm level in its promoter, thereby affecting its expression, resulting in a spurious correlation between mRNA of gene A and HyperZ/HypoZ. For gene B, the correlation of its expression with HyperZ/HypoZ is driven by the expression of another EE gene. For gene C, there is a direct influence between its expression and HyperZ/HypoZ. The partial correlation diagram depicts how these different models can be discriminated. Only for EE genes following model C would we see a significant partial correlation between their expression and HyperZ/HypoZ, whereas for genes of type A and B we would not. **b** A partial correlation network is derived for each tissue type and results summarized in a meta-analysis over the resulting networks. **c** Result of the causal network modeling meta-analysis (across all ten cancers) using partial correlation coefficients, identifying three EE genes whose expression patterns associate with HyperZ or HypoZ independently of other EE gene expression and their promoter DNAm levels

The correlations between EE gene expression and global DNAm indices may not represent direct effects (Fig. 5a). For example, widespread alterations in DNAm caused by an independent EE gene could alter the promoter DNAm level of a given EE gene, affecting its expression and resulting in an indirect correlation between its expression and the HyperZ/HypoZ indices. Thus, in order to pinpoint the more likely drivers of the global DNAm patterns in cancer, we devised a causal network modeling strategy implementing partial correlations [46] to remove indirect correlations (Fig. 5a). First, we implemented this strategy for each of the 18 EE genes separately. This revealed that, for most cases, the promoter DNAm of EE genes could not explain the observed associations between their expression and the global epigenetic instability indices with the exception of *PRDM5*, *SETBP1*, and *EYA4* in the case of HyperZ, and *DUSP1* and *TET3* in the case of HypoZ (Figure S6 in Additional file 1). Next, we implemented the causal network strategy using all previously identified 18 EE genes together in the inference procedure. Summarizing the inferred partial correlation (or direct influence) networks for each cancer type across all cancers (Fig. 5b) predicts that overexpression of *UHRF1* and *WHSC1* and underexpression of *CBX7* could be key drivers of cancer DNA hypermethylation and DNA hypomethylation, respectively (Fig. 5c).

### Candidate regulators of the cancer methylome affect DNAm levels at the same loci across different cancer types

**Fig. 6:**
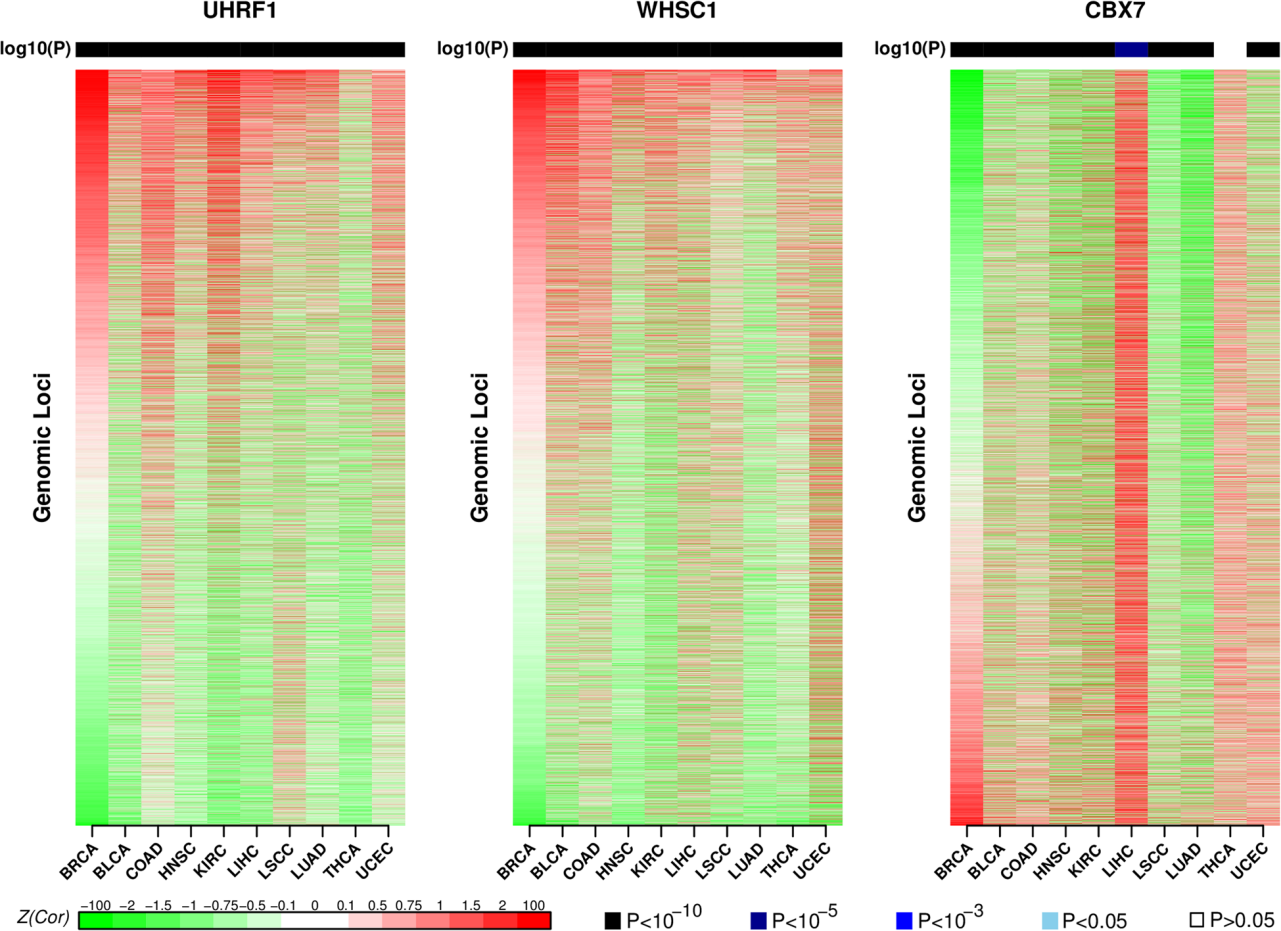
Correlation heatmaps of EE gene expression with DNAm levels of individual genomic loci across different cancer types. Heatmaps of correlation Fisher Z-statistics between the DNAm levels of ~140,000 genomic regions (~100,000 open sea plus ~40,000 CGI) and mRNA expression of the regulator, as indicated. For *UHRF1* and *WHSC1*, regions have been ranked from positive to negative correlations as determined in breast cancer, whereas for *CBX7*, regions have been ranked from negative to positive correlations. The same ranking is then used to depict the correlation statistics in the other cancer types. Above the heatmaps we give the *P* values corresponding to the Spearman rank correlation coefficient between the ranking in breast cancer and the ranking in every other cancer type

Finally, we asked if the predicted regulators (*UHRF1*, *WHSC1*, and *CBX7*) influence DNAm at the same genomic loci across cancer types. To this end, we ranked the genomic regions used previously to construct the HyperZ and HypoZ indices, according to their levels of association with the gene expression of either *UHRF1*, *WHSC1*, or *CBX7* (“Materials and methods”). Specifically, for each of the three regulators, we ranked the genomic loci according to the correlation statistic in one cancer type, and then asked if the correlations between DNAm and mRNA expression were similarly ranked in other cancer types (Fig. 6a). This showed that those genomic loci whose DNAm levels correlated most strongly with increased gene expression of *UHRF1* or *WHSC1* were also significantly highly ranked in most other cancer types (Fig. 6a, b; Additional file 3). Similarly, loci whose DNAm levels correlated most strongly with decreased gene expression of *CBX7* were also most significantly highly ranked in the other cancer types. Thus, we can see that these candidate master regulators tend to affect the same genomic loci, causing similar DNAm patterns, independent of tissue type.

Focusing on these top-ranked genomic loci also allowed us to check the effect sizes of the loci driving the global HyperZ and HypoZ indices as well as their correlations with the EE gene expression levels. Importantly, we observed that top ranked loci not only exhibited large effect sizes across tumors of a given type, but also between normal and cancer tissue, in support of their biological significance (Figures S7–S9 in Additional file 1).

## Discussion

The recent finding that genes encoding EEs are often found mutated in cancer, causing widespread changes to the DNA methylome and transcriptome, motivated us to perform a more in-depth exploration of the potential role of these EEs in cancer.

Our strategy was to perform an integrative pan-cancer-wide analysis of matched gene expression and DNAm data, in an attempt to identify EEs which display universal patterns of transcriptomic and epigenomic deregulation. Here, we did not consider mutation data for the following reasons. First, in a given cancer type, the frequency of mutation of a given EE is usually quite low. Second, functional disruption of an EE may be caused by a mechanism other than mutation, for instance by amplification or deletion, or by DNAm itself. Third, the effect of a mutation on gene function can be hard to predict. Thus, we hypothesized that, by anchoring the analysis on high-quality mRNA data of EE genes, this would allow us to discern broader and more universal patterns of epigenomic deregulation.

Confirming this, our meta-analysis clearly showed that several EE genes which are universally deregulated in cancer also appear to exhibit universal patterns of correlation with genome-wide DNAm levels. It is likely that these epigenetic genes constitute master regulators of the cancer DNA methylome. We initially identified 18 of such candidate regulators, with 11 representing putative oncogenes and seven representing putative tumor suppressors. In both cases, deregulation of the genes’ expression in cancer correlated positively with DNAm instability. A causal network modeling meta-analysis further filtered this list down to only three genes, predicting *UHRF1* and *WHSC1* to be oncogenic master regulators of the cancer DNA methylome, and *CBX7* to be a key tumor suppressor. Importantly, the genomic sites whose DNAm levels associated most strongly with expression of these genes were similar across different cancer types, further supporting the view that much of the deregulation of the DNA methylome obeys rules which transcend the type of cancer/tissue.

The prediction that *UHRF1* may be a key driver is noteworthy for various reasons. First, it has previously been implicated as an oncogene in many epithelial cancers, including, e.g., liver [47], prostate [48], breast [49], lung [50], colon [51–53] and bladder [54], and also hematological cancers [55]. Like *EZH2*, *UHRF1* also offers promising drug target potential [56]. Interestingly, *UHRF1* is a multi-faceted epigenetic regulator, whose main role is to recruit *DNMT1* during cell replication, and associations with both DNA hypermethylation and hypomethylation have been noted [47]. Our pan-cancer-wide analysis shows that expression of *UHRF1* correlates consistently with DNA hypermethylation across samples of a given cancer type, while it did not do so with DNA hypomethylation (Fig. 4). Furthermore, even though all major DNMTs were observed to be consistently overexpressed in cancer (Fig. 2), expression of these enzymes did not consistently correlate with the differences in global DNAm patterns between cancers. Thus, our study predicts that *UHRF1* may be an important driver of the aberrant DNA hypermethylation seen in cancer. Interestingly, *UHRF1* also plays a role in recruiting *HDAC1* [57], which we also found consistently overexpressed in cancer, and which correlated specifically with DNA hypomethylation (Fig. 4).

Our study also predicts important roles for *WHSC1*, a H3K36me writer, and *CBX7*, a H3K36me reader. The universal tumor suppressor role of the chromobox protein CBX7 is well supported by extensive literature in many different cancer types [38–40, 58–60]. Likewise, *WHSC1* has been previously implicated as an oncogene in numerous malignancies, including leukemias, liver, endometrial and ovarian cancer [61–66]. For instance, increased methyltransferase activity through point mutation in *WHSC1* has been shown to lead to widespread genome-wide increases in H3K36me2 and H3K36me3 marks [63]. Importantly, our data link putative deregulation of this histone mark via *WHSC1* to increased DNAm at promoter CGIs. Similarly, underexpression of *CBX7* appears to be associated with widespread inter-genic DNA hypomethylation in cancer, an entirely novel insight.

It is worth noting that the list of 11 candidate epigenetic oncogenes also included *TDG* (thymine DNA glycosylase) and *TET3*, both of which play key roles in the DNA demethylation pathway [67]. Consistent with this, we observed that overexpression of these two enzymes in cancers correlated specifically with an increased global DNA hypomethylation (Fig. 4). The list of epigenetic tumor suppressors included several histone methyltransferases, among them *PRDM2*/*5* and *SETBP1*. In agreement with previous studies, *PRDM2* has been found to be widely underexpressed in epithelial [68] and hematological cancers [69]. Mutations in *SETBP1* have been widely reported in hematological cancers. We observed that underexpression of either *PRDM2*, *PRDM5*, or *SETBP1* correlated with the HyperZ index, i.e., with increased DNAm at gene promoters.

All these results support the view that functional alterations of these EE genes are important determinants of cancer DNAm patterns. Although we here do not present experimental validation of these claims, our computational study does predict which of the EE genes are of particular interest to follow up experimentally. Another potential limitation of our study is the fact that the overwhelming majority of TCGA normal samples were collected adjacent to a matched tumor. Thus, by comparing with normal adjacent samples, our analysis may have missed important field defects. On the other hand, the results presented in this manuscript are unlikely to be affected by field defects. In fact, by comparing Illumina 450k DNAm data from 21 normal samples adjacent to a breast tumor with 30 normal samples from healthy women, we found that DNAm field defects are rare (Figure S10 in Additional file 1) and that global DNAm instability indices, such as HyperZ/HypoZ, are largely independent of which normal samples we use to construct them (Figure S11 in Additional file 1). As far as the RNA-Seq differential expression analysis is concerned, we verified that in the case of TCGA colon set (the only set for which we could find sufficient numbers of normals without matched cancer samples; Table S7 in Additional file 1), statistics of differential expression of all 212 EE genes were highly congruent, irrespective of whether paired or unpaired normals were used (Figure S12 in Additional file 1). Thus, field defects are very unlikely to have affected the global pan-cancer-wide meta-analysis results presented here.

Finally, our work has also shown that global levels of DNA hypermethylation and hypomethylation do not correlate that well within individual tumor samples: in most cancer types R^2^ values, although statistically significant, were around 0.1 or less. We interpret this finding as follows: a statistically significant correlation is expected given that tumors differ in terms of their level of normal cell contamination, and the HyperZ and HypoZ indices do correlate much more strongly if the normal samples are included. Second, several studies have reported that hypermethylated CGIs in cancer are often found immersed in large megabase scale blocks of hypomethylation [70, 71]. Thus, local correlations between these indices should translate to some level of correlation at the global scale. However, the relatively low R^2^ values also suggest that cancer hyper- and hypomethylation constitute independent processes in tumor progression, consistent with other reports [41–43]. We can reconcile all of these observations by noting that most studies did not consider the degree of quantitative change *across different tumors*. Thus, although in any given tumor a hypermethylated CGI will in general be contained within a large block of hypomethylation, the degree of hypermethylation and hypomethylation in that tumor may not necessarily correlate. Further supporting the view that global levels of cancer DNA hypermethylation and hypomethylation may be controlled by independent epigenetic processes, we also generally observed that EE genes correlating significantly with the HyperZ index did not do so with HypoZ, and vice versa. Interestingly, however, there were a few exceptions to this rule, which included *EZH2*, a component of the PRC2 complex, and *PCNA*, a well-known proliferation marker. Thus, the dynamics of DNAm change in cancer may have components which simultaneously cause hypermethylation of promoter CGIs and hypomethylation of open sea regions, whereas other components may act specifically to only cause promoter CGI hypermethylation or open sea hypomethylation. This is consistent with a recent study showing how differential expression patterns in cancer can arise due to widely different shapes of differential DNAm in and around a gene promoter’s region [72].

## Conclusions

Our analysis indicates that many EEs are not only aberrantly expressed in specific cancers, but that they also exhibit fairly universal patterns of deregulation across different cancer types, including common patterns of correlation with global DNAm levels. This supports the view that there are universal rules underlying the aberrant epigenomic architecture of cancer, i.e., rules which transcend cancer types. The master regulators of cancer DNAm patterns predicted here deserve intense experimental follow-up work and further computational study in order to help elucidate these rules.

## Materials and methods

### Collection and definition of an EE gene list

We used two excellent recent reviews [2, 3], as well as an additional literature search, to collate genes with roles in shaping the epigenome. Specifically, we collated genes encoding chromatin modification and remodeling enzymes, genes involved in the DNAm and/or DNA demethylation pathways, genes involved in histone modification, and genes involved in nucleosome positioning. A total of 212 chromatin modification/EE genes, including all main writers, readers, erasers and editors of the epigenome, from more than 20 gene families were collected (Table S1 in Additional file 1). Throughout this manuscript we refer to this class of 212 genes generally as epigenetic enzymes (EEs). Among the represented gene families were DNA (cytosine-5-)-methyltransferases (DNMTs), methyl-CpG-binding proteins (MBDs), isocitrate dehydrogenases (IDHs), ten-eleven translocation methylcytosine dioxygenases (TETs), zinc finger and BTB domain containing (ZBTBs), histone deacetylase (HDACs), histone acetyltransferases (HATs), lysine (K)-specific methyltransferases (KMTs), protein arginine N-methyltransferases (PRMTs), lysine (K)-specific demethylases (KDMs) and chromodomain helicase DNA binding proteins (CHDs) (see Table S1 in Additional file 1 for a full list).

### TCGA gene expression data

RNA-SeqV2 level 3 expression data, quantified as RSEM (RNA-Seq by expectation-maximization) were downloaded from TCGA. We downloaded the data for ten cancer types that had profiled sufficient numbers of cancer samples at both RNA-Seq and DNAm levels (Table S2 in Additional file 1). This included breast invasive carcinoma (BRCA) [32], bladder cancer (BLCA) [26], colon adenocarcinoma (COAD) [24], head and neck squamous cell carcinoma (HNSC) [23], kidney renal carcinoma (KIRC) [29], liver hepatocellular carcinoma (LIHC) [31], lung adenocarcinoma (LUAD) [25], lung squamous cell carcinoma (LUSC) [27], thyroid carcinoma (THCA) [28] and uterine corpus endometrial carcinoma (UCEC) [30]. The level 3 RNA-Seq data were processed further as follows: (i) zero-valued entries were replaced by the minimal positive value of the dataset; (ii) expression values were then logarithmically transformed (base 2) in order to regularize the data. Inter-sample variability and quality of the data were assessed using singular value decompositions (SVDs) [73] by checking that the top component of variation correlated with normal/cancer status. Before applying the SVD, the log-transformed expression values were first centered so that each gene had a mean zero across all samples. The number of significant components of variation was then inferred by using random matrix theory [74]. The significant components of variation were correlated to phenotypic and technical factors to assess the relative contributions of biological and technical variables to data variability and represented in a *P* value heatmap between components and factors.

### TCGA DNAm data

For the ten cancer types mentioned above, DNAm data generated with the Illumina Infinium HumanMethylation450 BeadChip array [75] were downloaded from TCGA data portal. The methylation level for each probe was obtained as the beta value, which was calculated from the intensity of methylated (M) and unmethylated (U) alleles: beta=Max(M,0)/[Max(M,0)+Max(U,0)+100]. The beta ranges from 0 (unmethylated) and 1 (fully methylated). Probes with missing data (i.e. NAs) in more than 70 % of the samples were removed. The rest of the probes with NAs were imputed using the k-nearest neighbors (knn) imputation procedure [76]. Subsequently, BMIQ was used to correct for the type II probe bias [77]. Data from each cancer type was then subjected to the same SVD quality control analysis, as done for gene expression.

### Erlangen Illumina 450k breast cancer DNAm data

Illumina 450k DNAm data for 30 normal samples (from healthy women), 21 normal samples adjacent to breast cancers, and 165 breast cancer samples were collected within the Bavarian Breast Cancer Cases and Controls Study 2. The Ethics Committee of the Medical Faculty, Friedrich-Alexander University approved the study (re. no. 4514) and all patients gave written informed consent. The study was done in adherence to the Declaration of Helsinki. Data are available in the Gene Expression Omnibus (accession number GSE69914). Raw data files were processed using the minfi, impute and BMIQ/ChAMP Bioconductor packages.

### Differential expression TCGA meta-analysis of EE genes across cancer

For each TCGA expression data set, we used moderated t-tests [78] to assess differential expression of approximately 20,000 genes between normal and corresponding cancer tissue, including the 212 EE genes. We note that we used all cancer samples and not just those with matched normal tissue. In view of the subsequent meta-analysis, we used relaxed nominal *P* value thresholds of 0.05 to declare statistical significance in each individual TCGA data set. We counted the number of EE genes which showed significant and consistent (i.e., same directionality) differential expression across at least eight of the ten cancer/tissue types. To assess the overall statistical significance of these counts, we also estimated the proportions of all human genome genes with significant overexpression and underexpression in each TCGA data set, thus obtaining “null” probabilities of overexpression (upregulation, *p*_*u*_) and underexpression (downregulated, *p*_*d*_). We observed that these probabilities did not vary much between cancer types (Table S3 in Additional file 1). Hence, we next estimated an average null probability for any given gene to be significantly upregulated or downregulated in cancer compared with normal tissue by taking the average of the corresponding probabilities across all cancer types. These average null probability estimates were ${\overline{p}}_u\approx 0.32$ and ${\overline{p}}_d\approx 0.34$. We then estimated the null probability that any given gene would be significantly upregulated (downregulated) in at least eight of the ten cancer types, using the binomial formula:

$$ p\left(nUP\ \ge\ 8\right)={\displaystyle \sum_{k=8}^{10}}\frac{10!}{k!\left(10-k\right)!}{\overline{p}}_u^k{\left(1-{\overline{p}}_u\right)}^{10-k} $$

$$ p\left(nDN\ \ge\ 8\right)={\displaystyle \sum_{k=8}^{10}}\frac{10!}{k!\left(10-k\right)!}{\overline{p}}_d^k{\left(1-{\overline{p}}_d\right)}^{10-k} $$

This yielded values of *p*(*nUP* ≥ 8) ≈ 0.003 and *p*(*nDN* ≥ 8) ≈ 0.004. Finally, given a pool of 212 random genes we can estimate the expected number which would be significantly upregulated (downregulated) in at least eight of the ten cancer types. This is given by a binomial distribution B(n,p) with (n = 212, *p* = 0.003) in the case of upregulation, and (n = 212, *p* = 0.004) for the case of downregulation. We find that *E*[*nUP* ≥ 8] ≈ 0.54(±0.73) and *E*[*nDN* ≥ 8] ≈ 0.89(±0.94), i.e., effectively we would expect only 1 of 212 genes to be explained by random chance. Finally, using the binomial distribution, we can estimate the statistical significance of the observed numbers of significant and consistently overexpressed and underexpressed EE genes. The observed numbers were 35 upregulated EE genes, and 27 downregulated EEs, which can’t be explained by random chance (*P* = 2e-53 for upregulated case, *P* = 9e-33 for downregulated case).

### Construction of epigenetic instability indices: HyperZ and HypoZ

In order to investigate whether the aberrant expression of EEs in a given cancer is associated with changes in the DNA methylome of that cancer, we first calculated “epigenetic instability indices” reflecting absolute deviations in DNAm in a given cancer sample, as assessed relative to normal samples from the same tissue type. We decided to construct two such indices, called HyperZ and HypoZ, to account for the potentially distinct mechanisms driving cancer DNA hypermethylation and DNA hypomethylation. The indices were constructed as follows: all CpGs in the genome were classified into different regional classes, according to whether they fall into open sea, CGI or shore/shelf regions, respectively [79]. All CpG sites within a regional class were then grouped together into regional clusters by using the boundedClusterMaker function of the *bumphunter* BioC package with a maximum cluster width of 1500 bp and a maximum gap of 500 bp between any two neighboring CpGs [80]. The methylation level for each regional cluster was defined as the average beta value of the CpGs within that cluster. For a given cluster/region, labeled *r*, in a given tumor sample *s*, we then computed a Z score, *Z*_*rs*_, reflecting the absolution deviation in DNAm of that region in the given cancer sample relative to all normal samples of the same tissue type. Specifically, let *μ*_*r*_^*(N)*^ and *σ*_*r*_^*(N)*^ denote the mean and standard deviation of the DNAm level of the regional cluster *r* over all the normal tissue samples. Then *Z*_*rs*_ was defined as ${Z}_{rs}=\frac{\beta_{rs}-{\mu}_r^{(N)}}{\sigma_r^{(N)}}$. Since regional clusters mapping to promoter CGIs are usually unmethylated in normal tissue, we only consider clusters for which the Z score in a given cancer sample is positive. Similarly, for open sea regional clusters, which are usually methylated in the normal tissue, we only consider clusters in a given cancer sample for which the Z score is negative, although we enforce positivity to ensure that the absolute deviation is taken into account. Specifically, the HyperZ index for a given cancer sample *s* was obtained as:

$$ Hyper{Z}_s=\frac{1}{n_r}{\displaystyle \sum_r^{n_r}{Z}_{rs}H\left({Z}_{rs}\right)} $$

where the summation is over all promoter CGI clusters and where *H*(*z*) denotes the Heaviside function: *H*(*z*) = 1 if *z > 0*, *H*(*z*) = 0 if *z* ≤ 0. Thus, only regions for which the Z score is positive contribute to the index, and the positivity of the index is guaranteed by definition. Similarly, the HypoZ index for a given cancer sample was estimated as:

$$ Hypor{Z}_s=\frac{1}{n_r}{\displaystyle \sum_r^{n_r}\left|{Z}_{rs}\right|H\left(-{Z}_{rs}\right)} $$

where the summation is now over all open sea regional clusters. The term involving the Heaviside function ensures that only regions with negative scores, i.e., hypomethylation from the methylated state, contribute. Taking the absolute value of the Z scores thus ensures that the index is always positive.

The HyperZ and HypoZ indices can be thought of as “epigenetic instability” indices in the sense that they measure global levels of absolute deviation in DNAm in a given cancer sample from a normal reference. The HyperZ index does so restricting to promoter CGIs and hence measures the overall level of cancer hypermethylation of these regions, whereas the HypoZ index reflects the overall absolute level of cancer hypomethylation in open sea regions.

In this manuscript we also use an alternative definition of the HyperZ and HypoZ indices, whereby the average is computed only over genomic regions, *r*, for which the Z score, *Z*_*rs*_, is significant (*P* < 0.05). This definition of the indices thus only uses significant regions. The correlation meta-analysis between RNA-Seq of EE genes and the HyperZ/HypoZ indices described below was performed using this latter definition of the indices, since for this definition, the HyperZ/HypoZ indices were less well correlated; thus, the two indices contain less redundant or more complementary information.

### Correlation meta-analysis of EE gene expression and epigenetic instability indices

Pearson correlation analysis was used to assess whether the expression of EEs is correlated with the HypoZ and HyperZ index from matched tumor samples. It is key to emphasize here that these correlations were computed only over tumor samples with matched RNA-Seq and DNAm data. Pearson correlation coefficients were transformed into Fisher Z-statistics $Z=0.5 \log \frac{1+PCC}{1-PCC}$ from which *P* values were then derived. Unadjusted *P* values <0.05 were deemed statistically significant. Once again the relaxed threshold was used because of the subsequent meta-analysis which would reassess statistical significance levels over all cancer types together. To assess statistical significance in the meta-analysis, we computed for each TCGA data set the fraction of genes (from all genes with RNA-Seq data) exhibiting significant positive and negative correlations with the HyperZ and HypoZ indices. This yielded four fractions/probabilities for each TCGA dataset, corresponding to positive correlations with HyperZ, negative correlations with HyperZ, positive correlations with HypoZ and negative correlations with HypoZ. From these fractions, we then computed an overall probability by averaging the corresponding probabilities over all cancer types. Denote these average probabilities as follows: ${\overline{p}}_{uu}$ for the average probability that a random gene is positively correlated with the HyperZ index; ${\overline{p}}_{du}$ for the average probability that a random gene is negatively correlated with the HyperZ index; ${\overline{p}}_{ud}$ for the case of positive correlations with HypoZ; and ${\overline{p}}_{dd}$ for the case of negative correlations with HypoZ. The specific estimates for these average probabilities were ${\overline{p}}_{uu}\approx 0.12,\ {\overline{p}}_{ud}\approx 0.16$ and ${\overline{p}}_{dd}\approx 0.25$. We then estimated the null probability that any given gene would be significantly positively (negatively) correlated with HyperZ in at least six of the ten cancer types, and similarly for HypoZ, using the binomial formulas:

$$ p\left(nUU\ \ge\ 6\right)={\displaystyle \sum_{k=6}^{10}}\frac{10!}{k!\left(10-k\right)!}{\overline{p}}_{uu}^k{\left(1-{\overline{p}}_{uu}\right)}^{10-k} $$

$$ p\left(nDU\ \ge\ 6\right)={\displaystyle \sum_{k=6}^{10}}\frac{10!}{k!\left(10-k\right)!}{\overline{p}}_{du}^k{\left(1-{\overline{p}}_{du}\right)}^{10-k} $$

$$ p\left(nUD\ \ge\ 6\right)={\displaystyle \sum_{k=6}^{10}}\frac{10!}{k!\left(10-k\right)!}{\overline{p}}_{ud}^k{\left(1-{\overline{p}}_{ud}\right)}^{10-k} $$

$$ p\left(nDD\ \ge\ 6\right)={\displaystyle \sum_{k=6}^{10}}\frac{10!}{k!\left(10-k\right)!}{\overline{p}}_{dd}^k{\left(1-{\overline{p}}_{dd}\right)}^{10-k} $$

This yielded values of *p*(*nUU* ≥ 6) ≈ 0.0004, *p*(*nDU* ≥ 6) ≈ 0.02, *p*(*nUD* ≥ 6) ≈ 0.002 and *p*(*DD* ≥ 6) ≈ 0.02. Finally, given a pool of 212 random genes we can estimate the expected number which would be significantly correlated (anti-correlated) with HyperZ or HypoZ in at least six of the ten cancer types. This is given by a binomial distribution B(n,p) with n = 212 and with p given by one of the four probabilities given above. We find that *E*[*nUU* ≥ 6] ≈ 0.54(±0.73) and *E*[*nDN* ≥ 8] ≈ 0.89(±0.94), i.e., effectively we would expect only 1 of 212 genes to be explained by random chance. Finally, using the binomial distribution, we can estimate the statistical significance of the observed numbers of significant and consistently overexpressed and underexpressed EE genes. The observed numbers were 35 upregulated EE genes, and 27 downregulated EE genes, which can’t be explained by random chance (*P* = 2e-53 for upregulated case, *P* = 9e-33 for downregulated case).

### Causal network modeling meta-analysis of EE genes

The differential expression meta-analysis and mRNA expression–HyperZ/HypoZ meta-analysis led to 18 EE genes, showing consistent differential expression and correlative patterns across cancer types. These 18 EE genes were then subjected to causal network modeling analysis in order to assess if the correlations of mRNA expression of these genes to the HyperZ/HypoZ indices is likely to be a direct effect, or if instead it is likely to be mediated by other factors (other EE genes or promoter DNAm levels of EE genes). Thus, the problem can be addressed by adopting a statistical method that can “silence” or remove correlations which are likely to be indirect. For this purpose, we used the framework of partial correlations/multivariate linear regressions [46]. Specifically, we conducted two separate analyses, one centered on individual EE genes, and another including all 18 EE genes in the model. In the first approach we estimated partial correlations between HyperZ/HypoZ and each EE gene’s expression level using the promoter DNAm level of the EE gene as a covariate. This allowed us to assess if the correlation between HyperZ/HypoZ and EE gene expression is independent of the EE gene’s DNAm promoter level. In the second approach, we used all other 17 EE gene expression as well as all 18 promoter DNAm levels as covariates, when estimating the partial correlation between a given EE gene’s expression with either the HyperZ or HypoZ index. This allowed us to assess if the correlation of an EE gene’s expression with HyperZ/HypoZ is not only independent of its promoter DNAm level, but also independent of the expression (and promoter DNAm) levels of the other 17 EE genes.

Application of this procedure in each cancer type led to a partial correlation network. We then constructed a consensus network over all ten cancer types, with edges defining significant and consistent partial correlations present in at least six of the ten cancer types.

### Correlation of genomic loci with EE gene expression

To assess if the same genomic loci are affected by a given EE gene, independently of cancer type, we adopted a genome-wide correlation approach. Specifically, we computed Pearson correlations between the DNAm level of any given region/cluster and the EE gene expression level, using only cancer samples to estimate the correlation. In the case of correlations with HyperZ, we only considered CGI-associated regions/clusters. In the case of correlations with HypoZ, we only considered open sea regions/clusters. Pearson correlations were transformed to Fisher Z-statistics. Spearman rank correlation and *P* values of the ranking obtained in each cancer type were used to evaluate consistency of rankings across cancer types.

## Additional files

Additional file 1:
**Supplementary Methods, Supplementary tables and all Supplementary figures, plus their associated legends/captions.**
Additional file 2:
**A Supplementary table listing differential expression t-statistics,** ***P*** **values and Benjamini-Hochberg adjusted** ***P*** **values for the 212 epigenetic enzymes across ten cancer types.**
Additional file 3:
**A Supplementary table listing the genomic loci whose DNA methylation levels correlate most strongly with mRNA expression of** ***UHRF1*** **,** ***WHSC1*** **and** ***CBX7*** **.** Loci have been ranked according to the average correlation across cancer types. The table gives the chromosome and genomic location of the region (or probe), the Pearson correlation coefficients and associated *P* values for each cancer type.

## Abbreviations

CGI: CpG island
DNAm: DNA methylation
EE: epigenetic enzyme
SVD: singular value decomposition
TCGA: The Cancer Genome Atlas

## References

[1] Hanahan D, Weinberg RA (2011). Hallmarks of cancer: the next generation. Cell.

[2] Shen H, Laird PW (2013). Interplay between the cancer genome and epigenome. Cell.

[3] Plass C, Pfister SM, Lindroth AM, Bogatyrova O, Claus R, Lichter P (2013). Mutations in regulators of the epigenome and their connections to global chromatin patterns in cancer. Nat Rev Genet.

[4] Fujimoto A, Totoki Y, Abe T, Boroevich KA, Hosoda F, Nguyen HH (2012). Whole-genome sequencing of liver cancers identifies etiological influences on mutation patterns and recurrent mutations in chromatin regulators. Nat Genet.

[5] Gui Y, Guo G, Huang Y, Hu X, Tang A, Gao S (2011). Frequent mutations of chromatin remodeling genes in transitional cell carcinoma of the bladder. Nat Genet.

[6] Jones DT, Jager N, Kool M, Zichner T, Hutter B, Sultan M (2012). Dissecting the genomic complexity underlying medulloblastoma. Nature.

[7] Yan XJ, Xu J, Gu ZH, Pan CM, Lu G, Shen Y (2011). Exome sequencing identifies somatic mutations of DNA methyltransferase gene DNMT3A in acute monocytic leukemia. Nat Genet.

[8] Turcan S, Rohle D, Goenka A, Walsh LA, Fang F, Yilmaz E (2012). IDH1 mutation is sufficient to establish the glioma hypermethylator phenotype. Nature.

[9] Noushmehr H, Weisenberger DJ, Diefes K, Phillips HS, Pujara K, Berman BP (2010). Identification of a CpG island methylator phenotype that defines a distinct subgroup of glioma. Cancer Cell.

[10] Gaidzik VI, Paschka P, Spath D, Habdank M, Kohne CH, Germing U (2012). TET2 mutations in acute myeloid leukemia (AML): results from a comprehensive genetic and clinical analysis of the AML study group. J Clin Oncol.

[11] Timp W, Feinberg AP (2013). Cancer as a dysregulated epigenome allowing cellular growth advantage at the expense of the host. Nat Rev Cancer.

[12] Hansen KD, Timp W, Bravo HC, Sabunciyan S, Langmead B, McDonald OG (2011). Increased methylation variation in epigenetic domains across cancer types. Nat Genet.

[13] Stadler SC, Allis CD (2012). Linking epithelial-to-mesenchymal-transition and epigenetic modifications. Semin Cancer Biol.

[14] Deaton AM, Bird A (2011). CpG islands and the regulation of transcription. Genes Dev.

[15] Ernst J, Kellis M (2010). Discovery and characterization of chromatin states for systematic annotation of the human genome. Nat Biotechnol.

[16] West AC, Johnstone RW (2014). New and emerging HDAC inhibitors for cancer treatment. J Clin Invest.

[17] Tian X, Zhang S, Liu HM, Zhang YB, Blair CA, Mercola D (2013). Histone lysine-specific methyltransferases and demethylases in carcinogenesis: new targets for cancer therapy and prevention. Curr Cancer Drug Targets.

[18] Ozdag H, Teschendorff AE, Ahmed AA, Hyland SJ, Blenkiron C, Bobrow L (2006). Differential expression of selected histone modifier genes in human solid cancers. BMC Genomics.

[19] Cohen AL, Piccolo SR, Cheng L, Soldi R, Han B, Johnson WE (2013). Genomic pathway analysis reveals that EZH2 and HDAC4 represent mutually exclusive epigenetic pathways across human cancers. BMC Med Genomics.

[20] Beck S, Rakyan VK (2008). The methylome: approaches for global DNA methylation profiling. Trends Genet.

[21] Varambally S, Cao Q, Mani RS, Shankar S, Wang X, Ateeq B (2008). Genomic loss of microRNA-101 leads to overexpression of histone methyltransferase EZH2 in cancer. Science.

[22] Asangani IA, Harms PW, Dodson L, Pandhi M, Kunju LP, Maher CA (2012). Genetic and epigenetic loss of microRNA-31 leads to feed-forward expression of EZH2 in melanoma. Oncotarget.

[23] Cancer Genome Atlas N (2015). Comprehensive genomic characterization of head and neck squamous cell carcinomas. Nature.

[24] Cancer Genome Atlas N (2012). Comprehensive molecular characterization of human colon and rectal cancer. Nature.

[25] Cancer Genome Atlas Research N (2014). Comprehensive molecular profiling of lung adenocarcinoma. Nature.

[26] Cancer Genome Atlas Research N (2014). Comprehensive molecular characterization of urothelial bladder carcinoma. Nature.

[27] Cancer Genome Atlas Research N (2012). Comprehensive genomic characterization of squamous cell lung cancers. Nature.

[28] Cancer Genome Atlas Research N (2014). Integrated genomic characterization of papillary thyroid carcinoma. Cell.

[29] Cancer Genome Atlas Research N (2013). Comprehensive molecular characterization of clear cell renal cell carcinoma. Nature.

[30] Kandoth C, Schultz N, Cherniack AD, Akbani R, Liu Y, Cancer Genome Atlas Research N (2013). Integrated genomic characterization of endometrial carcinoma. Nature.

[31] Kechavarzi B, Janga SC (2014). Dissecting the expression landscape of RNA-binding proteins in human cancers. Genome Biol.

[32] Koboldt DC, Fulton RS, McLellan MD, Schmidt H, Kalicki-Veizer J, McMichael JF (2012). Comprehensive molecular portraits of human breast tumours. Nature.

[33] Kondo Y (2014). Targeting histone methyltransferase EZH2 as cancer treatment. J Biochem.

[34] McCabe MT, Creasy CL (2014). EZH2 as a potential target in cancer therapy. Epigenomics.

[35] Kim K, Chadalapaka G, Lee SO, Yamada D, Sastre-Garau X, Defossez PA (2012). Identification of oncogenic microRNA-17-92/ZBTB4/specificity protein axis in breast cancer. Oncogene.

[36] Forzati F, Federico A, Pallante P, Colamaio M, Esposito F, Sepe R (2014). CBX7 gene expression plays a negative role in adipocyte cell growth and differentiation. Biol Open.

[37] Pallante P, Sepe R, Federico A, Forzati F, Bianco M, Fusco A (2014). CBX7 modulates the expression of genes critical for cancer progression. PLoS One.

[38] Forzati F, Federico A, Pallante P, Fedele M, Fusco A (2012). Tumor suppressor activity of CBX7 in lung carcinogenesis. Cell Cycle.

[39] Forzati F, Federico A, Pallante P, Abbate A, Esposito F, Malapelle U (2012). CBX7 is a tumor suppressor in mice and humans. J Clin Invest.

[40] Pallante P, Federico A, Berlingieri MT, Bianco M, Ferraro A, Forzati F (2008). Loss of the CBX7 gene expression correlates with a highly malignant phenotype in thyroid cancer. Cancer Res.

[41] Zhuang J, Jones A, Lee SH, Ng E, Fiegl H, Zikan M (2012). The dynamics and prognostic potential of DNA methylation changes at stem cell gene loci in women’s cancer. PLoS Genet.

[42] Frigola J, Sole X, Paz MF, Moreno V, Esteller M, Capella G (2005). Differential DNA hypermethylation and hypomethylation signatures in colorectal cancer. Hum Mol Genet.

[43] Yamashita K, Dai T, Dai Y, Yamamoto F, Perucho M (2003). Genetics supersedes epigenetics in colon cancer phenotype. Cancer Cell.

[44] Sorlie T, Tibshirani R, Parker J, Hastie T, Marron JS, Nobel A (2003). Repeated observation of breast tumor subtypes in independent gene expression data sets. Proc Natl Acad Sci U S A.

[45] Hu Z, Fan C, Oh DS, Marron JS, He XP, Qaqish BF (2006). The molecular portraits of breast tumors are conserved across microarray platforms. BMC Genomics.

[46] Opgen-Rhein R, Strimmer K (2007). From correlation to causation networks: a simple approximate learning algorithm and its application to high-dimensional plant gene expression data. BMC Syst Biol.

[47] Mudbhary R, Hoshida Y, Chernyavskaya Y, Jacob V, Villanueva A, Fiel MI (2014). UHRF1 overexpression drives DNA hypomethylation and hepatocellular carcinoma. Cancer Cell.

[48] Babbio F, Pistore C, Curti L, Castiglioni I, Kunderfranco P, Brino L (2012). The SRA protein UHRF1 promotes epigenetic crosstalks and is involved in prostate cancer progression. Oncogene.

[49] Yan F, Tan XY, Geng Y, Ju HX, Gao YF, Zhu MC (2011). Inhibition effect of siRNA-downregulated UHRF1 on breast cancer growth. Cancer Biother Radiopharm.

[50] Daskalos A, Oleksiewicz U, Filia A, Nikolaidis G, Xinarianos G, Gosney JR (2011). UHRF1-mediated tumor suppressor gene inactivation in nonsmall cell lung cancer. Cancer.

[51] Wang F, Yang YZ, Shi CZ, Zhang P, Moyer MP, Zhang HZ (2012). UHRF1 promotes cell growth and metastasis through repression of p16(ink(4)a) in colorectal cancer. Ann Surg Oncol.

[52] Du Z, Song J, Wang Y, Zhao Y, Guda K, Yang S (2010). DNMT1 stability is regulated by proteins coordinating deubiquitination and acetylation-driven ubiquitination. Sci Signal.

[53] Sabatino L, Fucci A, Pancione M, Carafa V, Nebbioso A, Pistore C (2012). UHRF1 coordinates peroxisome proliferator activated receptor gamma (PPARG) epigenetic silencing and mediates colorectal cancer progression. Oncogene.

[54] Unoki M, Kelly JD, Neal DE, Ponder BA, Nakamura Y, Hamamoto R (2009). UHRF1 is a novel molecular marker for diagnosis and the prognosis of bladder cancer. Br J Cancer.

[55] Guan D, Factor D, Liu Y, Wang Z, Kao HY (2013). The epigenetic regulator UHRF1 promotes ubiquitination-mediated degradation of the tumor-suppressor protein promyelocytic leukemia protein. Oncogene.

[56] Unoki M, Brunet J, Mousli M (2009). Drug discovery targeting epigenetic codes: the great potential of UHRF1, which links DNA methylation and histone modifications, as a drug target in cancers and toxoplasmosis. Biochem Pharmacol.

[57] Unoki M, Nishidate T, Nakamura Y (2004). ICBP90, an E2F-1 target, recruits HDAC1 and binds to methyl-CpG through its SRA domain. Oncogene.

[58] Shinjo K, Yamashita Y, Yamamoto E, Akatsuka S, Uno N, Kamiya A (2014). Expression of chromobox homolog 7 (CBX7) is associated with poor prognosis in ovarian clear cell adenocarcinoma via TRAIL-induced apoptotic pathway regulation. Int J Cancer.

[59] Kim HY, Park JH, Won HY, Lee JY, Kong G (2015). CBX7 inhibits breast tumorigenicity through DKK-1-mediated suppression of the Wnt/beta-catenin pathway. FASEB J.

[60] Braun CJ, Hemann MT (2013). Unraveling tumor suppressor networks with in vivo RNAi. Cell Stem Cell.

[61] Hudlebusch HR, Skotte J, Santoni-Rugiu E, Zimling ZG, Lees MJ, Simon R (2011). MMSET is highly expressed and associated with aggressiveness in neuroblastoma. Cancer Res.

[62] Hudlebusch HR, Santoni-Rugiu E, Simon R, Ralfkiaer E, Rossing HH, Johansen JV (2011). The histone methyltransferase and putative oncoprotein MMSET is overexpressed in a large variety of human tumors. Clin Cancer Res.

[63] Oyer JA, Huang X, Zheng Y, Shim J, Ezponda T, Carpenter Z (2014). Point mutation E1099K in MMSET/NSD2 enhances its methyltranferase activity and leads to altered global chromatin methylation in lymphoid malignancies. Leukemia.

[64] Yang S, Zhang Y, Meng F, Liu Y, Xia B, Xiao M (2013). Overexpression of multiple myeloma SET domain (MMSET) is associated with advanced tumor aggressiveness and poor prognosis in serous ovarian carcinoma. Biomarkers.

[65] Xiao M, Yang S, Chen J, Ning X, Guo L, Huang K (2013). Overexpression of MMSET in endometrial cancer: a clinicopathologic study. J Surg Oncol.

[66] Zhou P, Wu LL, Wu KM, Jiang W, Li JD, Zhou LD (2013). Overexpression of MMSET is correlation with poor prognosis in hepatocellular carcinoma. Pathol Oncol Res.

[67] Hu X, Zhang L, Mao SQ, Li Z, Chen J, Zhang RR (2014). Tet and TDG mediate DNA demethylation essential for mesenchymal-to-epithelial transition in somatic cell reprogramming. Cell Stem Cell.

[68] Steele-Perkins G, Fang W, Yang XH, Van Gele M, Carling T, Gu J (2001). Tumor formation and inactivation of RIZ1, an Rb-binding member of a nuclear protein-methyltransferase superfamily. Genes Dev.

[69] Xie W, Li X, Chen X, Huang S (2010). Decreased expression of PRDM2 (RIZ1) and its correlation with risk stratification in patients with myelodysplastic syndrome. Br J Haematol.

[70] Timp W, Bravo HC, McDonald OG, Goggins M, Umbricht C, Zeiger M (2014). Large hypomethylated blocks as a universal defining epigenetic alteration in human solid tumors. Genome Med.

[71] Berman BP, Weisenberger DJ, Aman JF, Hinoue T, Ramjan Z, Liu Y (2012). Regions of focal DNA hypermethylation and long-range hypomethylation in colorectal cancer coincide with nuclear lamina-associated domains. Nat Genet.

[72] Vanderkraats ND, Hiken JF, Decker KF, Edwards JR (2013). Discovering high-resolution patterns of differential DNA methylation that correlate with gene expression changes. Nucleic Acids Res.

[73] Teschendorff AE, Menon U, Gentry-Maharaj A, Ramus SJ, Gayther SA, Apostolidou S (2009). An epigenetic signature in peripheral blood predicts active ovarian cancer. PLoS One.

[74] Teschendorff AE, Zhuang J, Widschwendter M (2011). Independent surrogate variable analysis to deconvolve confounding factors in large-scale microarray profiling studies. Bioinformatics.

[75] Sandoval J, Heyn H, Moran S, Serra-Musach J, Pujana MA, Bibikova M (2011). Validation of a DNA methylation microarray for 450,000 CpG sites in the human genome. Epigenetics.

[76] Troyanskaya O, Cantor M, Sherlock G, Brown P, Hastie T, Tibshirani R (2001). Missing value estimation methods for DNA microarrays. Bioinformatics.

[77] Teschendorff AE, Marabita F, Lechner M, Bartlett T, Tegner J, Gomez-Cabrero D (2013). A beta-mixture quantile normalization method for correcting probe design bias in Illumina Infinium 450 k DNA methylation data. Bioinformatics.

[78] Smyth GK (2004). Linear models and empirical bayes methods for assessing differential expression in microarray experiments. Stat Appl Genet Mol Biol.

[79] Jiao Y, Widschwendter M, Teschendorff AE (2014). A systems-level integrative framework for genome-wide DNA methylation and gene expression data identifies differential gene expression modules under epigenetic control. Bioinformatics.

[80] Jaffe AE, Murakami P, Lee H, Leek JT, Fallin MD, Feinberg AP (2012). Bump hunting to identify differentially methylated regions in epigenetic epidemiology studies. Int J Epidemiol.

