# Supplementary material for: An integrative pan-cancer-wide analysis of epigenetic enzymes reveals universal patterns of epigenomic deregulation in cancer
Source: Genome Biol. 2015 Jul 14;16(1):140. doi: 10.1186/s13059-015-0699-9 (PMC4501092; doi:10.1186/s13059-015-0699-9)
Supplement: Additional file 1: — Supplementary Methods, Supplementary tables and all Supplementary figures, plus their associated legends/captions. [file 13059_2015_699_MOESM1_ESM.pdf]

Additional data file 1

An integrative pan-cancer-wide analysis of epigenetic enzymes reveals universal patterns of epigenomic deregulation in cancer

Yang Zhen [1], Allison Jones[2], Martin Widschwendter[2] and Andrew E. Teschendorff [1,3,*]

*Corresponding author: Andrew E. Teschendorff- a.teschendorff@ucl.ac.uk

(1) Key Laboratory of Computational Biology, CAS-MPG Partner Institute for Computational Biology, 320 Yue Yang Road, Shanghai 200031, China. (2) Department of Women's Cancer, University College London, 74 Huntley Street, London WC1E 6AU, United Kingdom. (3) Statistical Cancer Genomics, Paul O'Gorman Building, UCL Cancer Institute, University College London, 72 Huntley Street, London WC1E 6BT, United Kingdom.

This document contains Supplementary Methods, Figures and Tables.

SUPPLEMENTARY METHODS

Collection and definition of an Epigenetic Enzyme (EE) gene list:

We used two excellent recent reviews [1, 2], as well as an additional literature search, to collate genes with roles in shaping the epigenome. Specifically, we collated genes encoding chromatin modification and remodelling enzymes, genes involved in the DNA methylation and/or DNA-demethylation pathways, genes involved in histone modification, and genes involved in nucleosome positioning. A total of 212 chromatin modification/epigenetic enzyme genes, including all main writers, readers, erasers and editors of the epigenome, from more than twenty gene families were collected (**Table S1**). Throughout this manuscript we refer to this class of 212 genes, generally as Epigenetic Enzymes (EE). Among the represented gene families were DNA (cytosine-5-)-methyltransferases (DNMTs), Methyl-CpG-Binding Proteins (MBDs), Isocitrate dehydrogenases (IDHs), Ten-eleven translocation methylcytosine dioxygenases (TETs), Zinc finger and BTB domain containing (ZBTBs), Histone deacetylase (HDACs), Histone acetyltransferases (HATs), lysine (K)-specific methyltransferase (KMTs),

protein arginine N-methyltransferase (PRMTs), lysine (K)-specific demethylase (KDMs) and chromodomain helicase DNA binding protein (CHDs) (see **Table S1** for full list**).**

Gene expression data (TCGA):

RNA-SeqV2 level-3 expression data, quantified as RSEM (RNA-Seq by Expectation-Maximization) were downloaded from The Cancer Genome Atlas (TCGA). We downloaded the data for 10 cancer types that had profiled sufficient numbers of cancer samples at both RNAseq and DNA methylation levels (**Table S2**). This included breast invasive carcinoma (BRCA) [3], bladder cancer (BLCA) [4], colon adenocarcinoma (COAD) [5], head and neck squamous cell carcinoma (HNSC) [6], kidney renal carcinoma (KIRC) [7], liver hepatocellular carcinoma (LIHC) [8], lung adenocarcinoma (LUAD) [9], lung squamous cell carcinoma (LUSC) [10], thyroid carcinoma (THCA) [11] and uterine corpus endometrial carcinoma (UCEC) [12]. The level-3 RNA-Seq data was processed further as follows: (i) zero-valued entries were replaced by the minimal positive value of the dataset, (ii) expression values were then logarithmically transformed (base 2) in order to regularize the data. Inter-sample variability and quality of the data was assessed using Singular Value Decompositions (SVD) [13], by checking that the top component of variation correlated with normal/cancer status. Before applying the SVD, the log transformed expression values were first centered so that each gene had a mean zero across all samples. The number of significant components of variation was then inferred by using Random Matrix Theory [14]. The significant components of variation were correlated to phenotypic and technical factors to assess the relative contributions of biological and technical variables to data variability and represented in a P-value heatmap between components and factors.

DNA methylation data (TCGA):

For the 10 cancer types mentioned above, DNA methylation data generated with the Illumina Infinium HumanMethylation450 BeadChip array [15] were downloaded from the TCGA data portal. The methylation level for each probe was obtained as the beta value, which was calculated from the intensity of methylated (M) and unmethylated (U) alleles: beta=Max(M,0)/[Max(M,0)+Max(U,0)+100]. The beta ranges from 0 (unmethylated) and 1(fully methylated). Probes with missing data in more than 70% of the samples were removed. The rest of probes with NA's were imputed using the k-nearest neighbors (knn) imputation procedure [16]. Subsequently, BMIQ was used to correct for the type II probe bias [17]. Data from each cancer type was then subjected to the same SVD quality control analysis, as done for gene expression.

Erlangen Illumina 450k breast cancer DNA methylation data:

Illumina 450k DNA methylation data for 30 normal samples (from healthy women), 21 normal samples adjacent to breast cancers, and 165 breast cancer samples were collected within the Bavarian Breast Cancer Cases and Controls Study 2. The Ethics Committee of the Medical Faculty, Friedrich-Alexander University approved the study (Re. No. 4514) and all patients gave written informed consent. The study was done in adherence to the Declaration of Helsinki. Data are available in GEO (accession number GSE69914 to be determined). Raw data files were processed using the minfi, impute and BMIQ/ChAMP Bioconductor packages.

Differential expression Meta-Analysis of EE genes across cancer

For each TCGA expression data set, we used moderated t-tests [18] to assess differential expression (DE) of approximately 20000 genes between normal and corresponding cancer tissue, including the 212 EE genes. We note that we used all cancer samples and not just those with matched normal tissue. In view of the subsequent meta-analysis, we used relaxed nominal P-value thresholds of 0.05 to declare statistical significance in each individual TCGA data set. We counted the number of EE genes which showed significant and consistent (i.e. same directionality) differential expression across at least 8 of the 10 cancer/tissue types. To assess the overall statistical significance of these counts, we also estimated the proportions of all human genome genes with significant overexpression and underexpression in each TCGA data set, thus obtaining "null" probabilities of overexpression (upregulation, p_u) and underexpression (downregulated, p_d). We observed that these probabilities did not vary much between cancer types. Hence, we next estimated an average null probability for any given gene to be significantly upregulated or downregulated in cancer compared to normal tissue, by taking the average of the corresponding probabilities across all cancer types. These average null probability estimates were $\bar{p}_u \approx 0.32$ and $\bar{p}_d \approx 0.34$. We then estimated the null probability that any given gene would be significantly upregulated (downregulated) in at least 8 of the 10 cancer types, using the Binomial formula:

$$p(nUP \geq 8) = \sum_{k=8}^{10} \frac{10!}{k!\,(10-k)!} \bar{p}_u^k \,(1-\bar{p}_u)^{10-k}$$

$$p(nDN \geq 8) = \sum_{k=8}^{10} \frac{10!}{k!\,(10-k)!} \bar{p}_d^k \,(1-\bar{p}_d)^{10-k}$$

This yielded values of $p(nUP \geq 8) \approx 0.003$ and $p(nDN \geq 8) \approx 0.004$. Finally, given a pool of 212 random genes we can estimate the expected number which would be significantly

upregulated (downregulated) in at least 8 of the 10 cancer types. This is given by a Binomial distribution B(n,p) with (n=212,p=0.003) in the case of upregulation, and (n=212,p=0.004) for the case of downregulation. We find that $E[nUP \geq 8] \approx 0.54(\pm 0.73)$ and $E[nDN \geq 8] \approx 0.89(\pm 0.94)$, i.e. effectively we would expect only 1 of 212 genes to be explained by random chance. Finally, using the Binomial distribution, we can estimate the statistical significance of the observed numbers of significant and consistently overexpressed and underexpressed EE genes. The observed numbers were 35 upregulated EE genes, and 27 downregulated EE's, which can't be explained by random chance (P=2e-53 for upregulated case, P=9e-33 for downregulated case).

Construction of Epigenetic Instability Indices: HyperZ and HypoZ

In order to investigate whether the aberrant expression of epigenetic enzymes in a given cancer is associated with changes in the DNA methylome of that cancer, we first calculated "Epigenetic Instability Indices" reflecting deviations in DNA methylation in a given cancer sample, as assessed relative to normal samples from the same tissue type. We decided to construct two such indices, called HyperZ and HypoZ, to account for the potentially distinct mechanisms driving cancer DNA hypermethylation and DNA hypomethylation. The indices were constructed as follows: all CpGs in the genome were classified into different regional classes, according to whether they fall into Open Sea, CpG Island or Shore/Shelve regions, respectively [19]. All CpG sites within a regional class were then grouped together into regional clusters, by using the boundedClusterMaker function of the *bumphunter* BioC package with a maximum cluster width of 1,500bp and a maximum gap of 500bp between any two neighboring CpGs [20]. The methylation level for each regional cluster was defined as the average beta value of the CpGs within that cluster. For a given cluster/region, labeled r, in a given tumour sample s, we then computed a Z-score, Z_{rs} , reflecting the absolution deviation in DNA methylation of that region in the given cancer sample relative to all normal samples of the same tissue type. Specifically, let $\mu_r^{(N)}$ and $\sigma_r^{(N)}$ denote the mean and standard deviation of the DNA methylation level of the regional cluster r over all the normal tissue samples. Then Z_{rs} was defined as $Z_{rs} = \frac{\beta_{rs} - \mu_r^{(N)}}{\sigma_r^{(N)}}$. Since regional clusters mapping to promoter CGIs are usually unmethylated in normal tissue, we only consider clusters for which the Z-score in a given cancer sample is positive. Similarly, for open sea regional clusters, which are usually methylated in the normal tissue, we only consider clusters in a given cancer sample for which the Z-score is negative, although we enforce positivity to ensure that the absolute deviation is taken into account. Specifically, the HyperZ index for a given cancer sample s was obtained as

$$HyperZ_s = \frac{1}{n_r}\sum_{r}^{n_r} Z_{rs} H(Z_{rs})$$

where the summation is over all promoter CpG island clusters and where $H(z)$ denotes the Heaviside function: $H(z)=1$ if $z > 0$, $H(z)=0$ if $z \leq 0$. Thus, only regions for which the Z-score is positive contribute to the index, and the positivity of the index is guaranteed by definition. Similarly, the HypoZ index for a given cancer sample was estimated as

$$HypoZ_s = \frac{1}{n_r}\sum_{r}^{n_r} |Z_{rs}| H(-Z_{rs})$$

where the summation is now over all open sea regional clusters. The term involving the Heaviside function ensures that only regions with negative scores, i.e. hypomethylation from the methylated state, contribute. Taking the absolute value of the Z-scores thus ensures that the index is always positive.

The HyperZ and HypoZ indices can be thought of as “epigenetic instability” indices in the sense that they measure global levels of absolute deviation in DNA methylation in a given cancer samples from a normal reference. The HyperZ index does so restricting to promoter CpG islands and hence measures the overall level of cancer hypermethylation of these regions, whereas the HypoZ index reflects the overall absolute level of cancer hypomethylation in open sea regions.

In this manuscript we also use an alternative definition of the HyperZ and HypoZ indices, whereby the average is computed only over genomic regions, r, for which the Z-score, Z_{rs} , is significant (P<0.05). This definition of the indices thus only uses significant regions. The correlation meta-analysis between RNA-seq of EE genes and the HyperZ/HypoZ indices, described below was performed using this latter definition of the indices, since for this definition, the HyperZ/HypoZ indices were less well correlated, thus the two indices contain less redundant or more complementary information.

Correlation Meta-Analysis of EE gene expression and Epigenetic Instability Indices

Pearson correlation analysis was used to assess whether the expression of EEs is correlated with the HypoZ-and HyperZ-index from matched tumor samples. It is key to emphasize here that these correlations were computed only over tumour samples with matched RNA-Seq and DNAm data. Pearson correlation coefficients (PCC) were transformed into Fisher Z-statistics $Z = 0.5 \log\frac{1+PCC}{1-PCC}$ from which P-values were then derived. Unadjusted P-values < 0.05 were deemed statistically significant. Once again the relaxed threshold was used because of the subsequent meta-analysis which would reassess statistical significance levels over all cancer

types together. To assess statistical significance in the meta-analysis, we computed for each TCGA data set, the fraction of genes (from all genes with RNA-Seq data) exhibiting significant positive and negative correlations with the HyperZ and HypoZ indices. This yielded 4 fractions/probabilities for each TCGA dataset, corresponding to positive correlations with HyperZ, negative correlations with HyperZ, positive correlations with HypoZ and negative correlations with HypoZ. From these fractions, we then computed an overall probability by averaging the corresponding probabilities over all cancer types. Denote these average probabilities as follows: $\bar{p}_{uu}$ for the average probability that a random gene is positively correlated with the HyperZ index, $\bar{p}_{du}$ for the average probability that a random gene is negatively correlated with the HyperZ index, $\bar{p}_{ud}$ for the case of positive correlations with HypoZ, and $\bar{p}_{dd}$ for the case of negative correlations with HypoZ. The specific estimates for these average probabilities were $\bar{p}_{uu} \approx 0.12$, $\bar{p}_{du} \approx 0.25$, $\bar{p}_{ud} \approx 0.16$ and $\bar{p}_{dd} \approx 0.25$.

We then estimated the null probability that any given gene would be significantly positively (negatively) correlated with HyperZ in at least 6 of the 10 cancer types, and similarly for HypoZ, using the Binomial formulas:

$$p(nUU \geq 6) = \sum_{k=6}^{10} \frac{10!}{k!\,(10-k)!} \bar{p}_{uu}^{k} \,(1 - \bar{p}_{uu})^{10-k}$$

$$p(nDU \geq 6) = \sum_{k=6}^{10} \frac{10!}{k!\,(10-k)!} \bar{p}_{du}^{k} \,(1 - \bar{p}_{du})^{10-k}$$

$$p(nUD \geq 6) = \sum_{k=6}^{10} \frac{10!}{k!\,(10-k)!} \bar{p}_{ud}^{k} \,(1 - \bar{p}_{ud})^{10-k}$$

$$p(nDD \geq 6) = \sum_{k=6}^{10} \frac{10!}{k!\,(10-k)!} \bar{p}_{dd}^{k} \,(1 - \bar{p}_{dd})^{10-k}$$

This yielded values of $p(nUU \geq 6) \approx 0.0004$, $p(nDU \geq 6) \approx 0.02$, $p(nUD \geq 6) \approx 0.002$ and $p(DD \geq 6) \approx 0.02$. Finally, given a pool of 212 random genes we can estimate the expected number which would be significantly correlated (anti-correlated) with HyperZ or HypoZ in at least 6 of the 10 cancer types. This is given by a Binomial distribution B(n,p) with n=212 and with p given by one of the four probabilities given above. We find that $E[nUU \geq 6] \approx 0.54(\pm 0.73)$ and $E[nDN \geq 8] \approx 0.89(\pm 0.94)$, i.e. effectively we would expect only 1 of 212 genes to be explained by random chance. Finally, using the Binomial distribution, we can estimate the statistical significance of the observed numbers of significant and consistently overexpressed and underexpressed EE genes. The observed numbers were 35 upregulated EE genes, and 27 downregulated EE's, which can't be explained by random chance (P=2e-53 for upregulated case, P=9e-33 for downregulated case).

Causal Network Modelling Meta-Analysis of EE genes

The differential expression meta-analysis and mRNA expression – HyperZ/HypoZ meta-analysis led to 18 EE genes, showing consistent differential expression and correlative patterns across cancer types. These 18 EE genes were then subjected to causal network modelling analysis in order to assess if the correlations of mRNA expression of these genes to the HyperZ/HypoZ indices is likely to be a direct effect, or if instead it is likely to be mediated by other factors (other EE genes or promoter DNAm levels of EE genes). Thus, the problem can be addressed by adopting a statistical method that can "silence" or remove correlations which are likely to be indirect. For this purpose, we used the framework of partial correlations/multivariate linear regressions [21]. Specifically, we conducted two separately analyses, one centred on individual EE genes, and another, including all 18 EE genes in the model. In the first approach we estimated partial correlations between HyperZ/HypoZ and each EE gene's expression level using the promoter DNAm level of the EE gene as a covariate. This allowed us to assess if the correlation between HyperZ/HypoZ and EE gene expression is independent of the EE gene's DNAm promoter level. In the second approach, we used all other 17 EE gene expression as well as all 18 promoter DNAm levels as covariates, when estimating the partial correlation between a given EE gene's expression with either the HyperZ or HypoZ index. This allowed us to assess if the correlation of a EE gene's expression with HyperZ/HypoZ is not only independent of its promoter DNAm level, but also independent from the expression (and promoter DNAm) levels of the other 17 EE genes.

Application of this procedure in each cancer-type led to a partial correlation network. We then constructed a consensus network over all 10 cancer types, with edges defining significant and consistent partial correlations present in at least 6 of the 10 cancer types.

Correlation of genomic loci with EE gene expression

To assess if the same genomic loci are affected by a given EE gene, independently of cancer type, we adopted a genome-wide correlation approach. Specifically, we computed Pearson correlations between the DNAm level of any given region/cluster and the EE gene expression level, using only cancer samples to estimate the correlation. In the case of correlations with HyperZ, we only considered CpG island associated regions/clusters. In the case of correlations with HypoZ, we only considered opensea regions/clusters. Pearson correlations were transformed to Fisher Z-statistics. Spearman rank correlation and P-values of the ranking obtained in each cancer type were used to evaluate consistency of rankings across cancer types.

SUPPLEMENTARY FIGURES

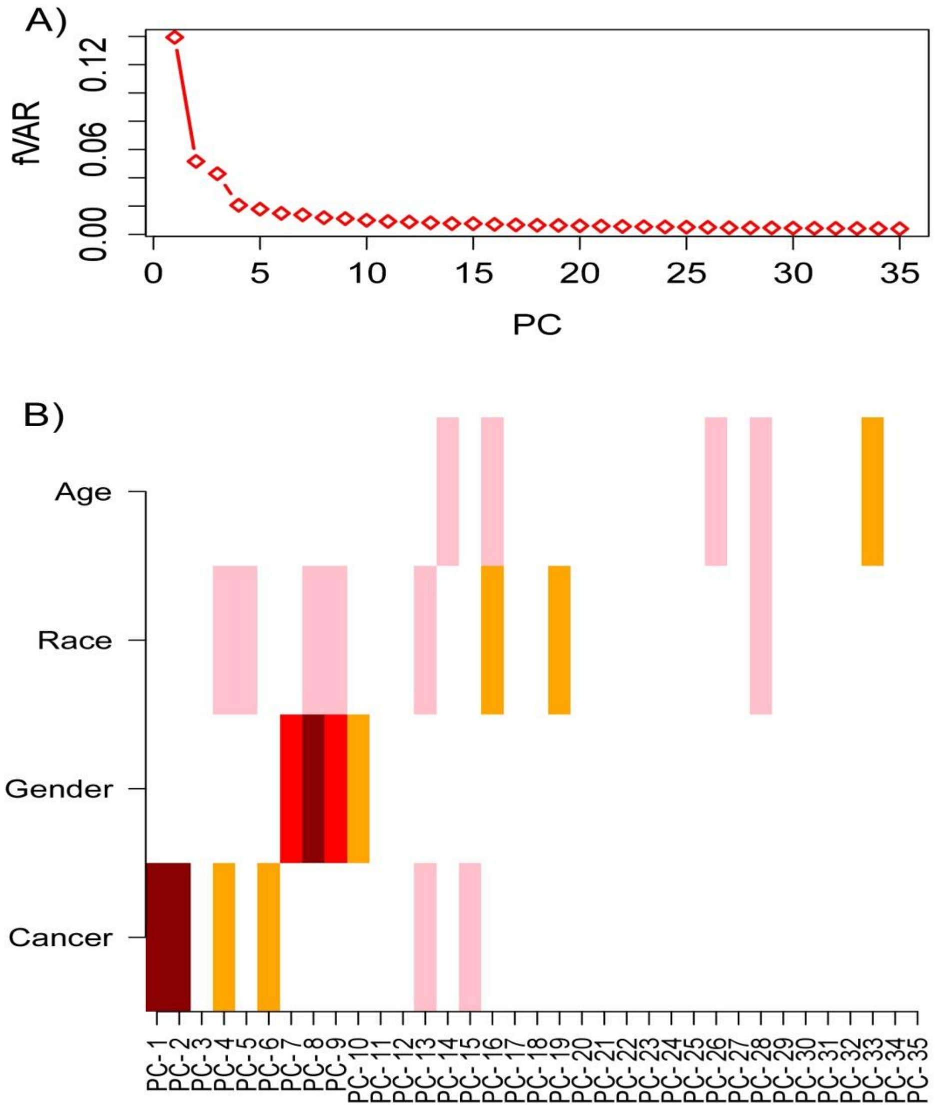

Figure.S1: Quality Control (QC) Analysis of RNA-Seq data from the TCGA. Illustrated is the example of colon cancer which passed QC. Top panel plots the fractional variation of the total data variation accounted for by each of the top-ranked and significant singular vectors (principal components-PC) from the SVD analysis. The number of significantly variable singular vectors was determined by Random Matrix Theory (RMT) analysis [14]. Lower panel depicts a heatmap of P-values of association between each singular vector/principal component and phenotypic and technical factors. Color Codes: Dark-red (P<1e-10), Red (P<1e-5), Orange (P<0.001), Pink (P<0.05), White (P > 0.05). As we can see, the top PC correlates most strongly with normal/cancer status, as we would expect. Only those cancer-types for which the top-PC correlated unambiguously with normal/cancer status (for both RNA-Seq and 450k DNA methylation) were used in this study.

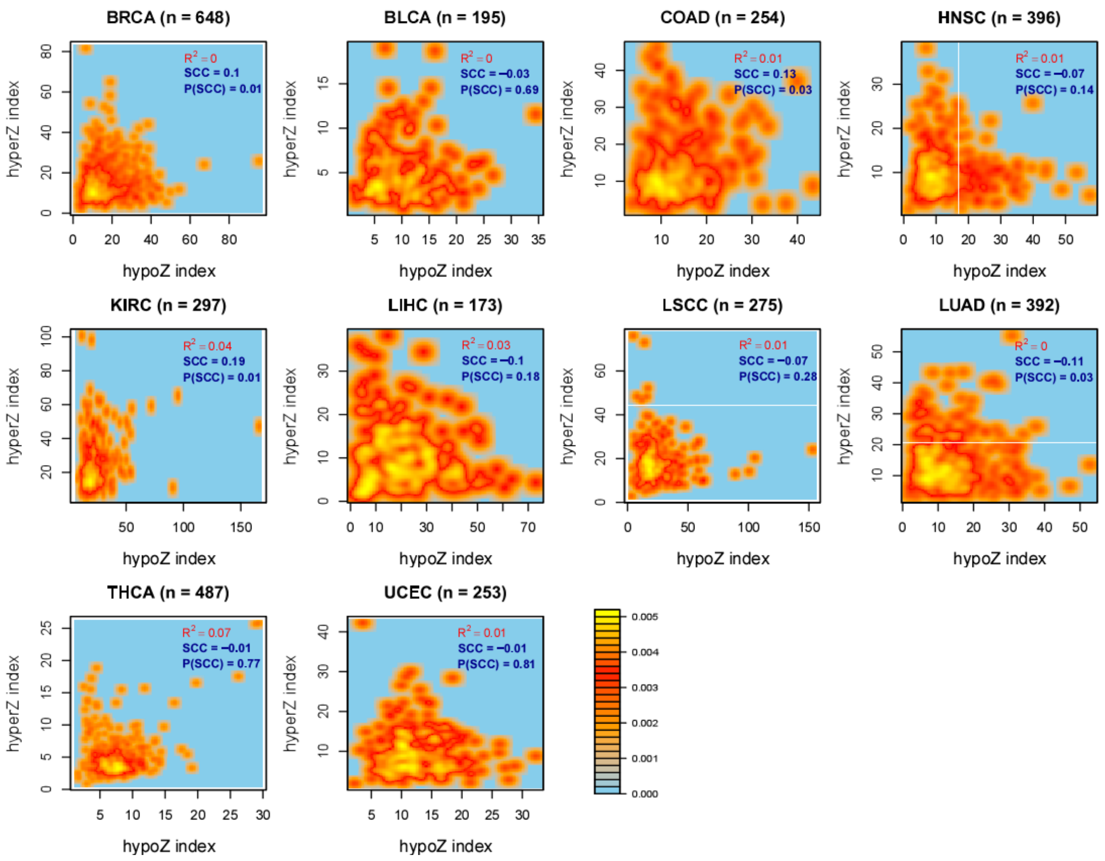

Figure.S2: For each cancer type, we display two-dimensional density plots (bright yellow indicates highest density) illustrating the distribution of tumours in the plane defined by the HyperZ and HypoZ indices. The number of tumours is given above each panel. For each cancer type, we provide the Spearman Correlation Coefficient (SCC), its P-value, as well as the R2-value for a linear regression. Cancer types are abbreviated as in Fig.2. We note that the HyperZ and HypoZ indices used in this plot were defined by restricting to genomic regions which showed significant Z-scores.

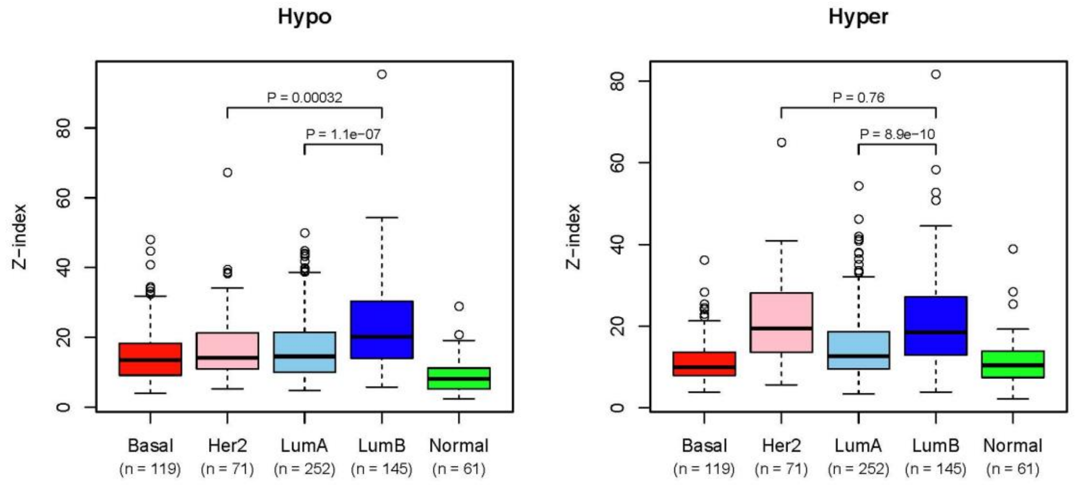

Figure.S3: Boxplot of HyperZ and HypoZ DNA methylation instability indices across the breast cancers of the TCGA study, stratified according to the PAM50 intrinsic subtype [22]. P-values are from a Wilcoxon rank sum test.

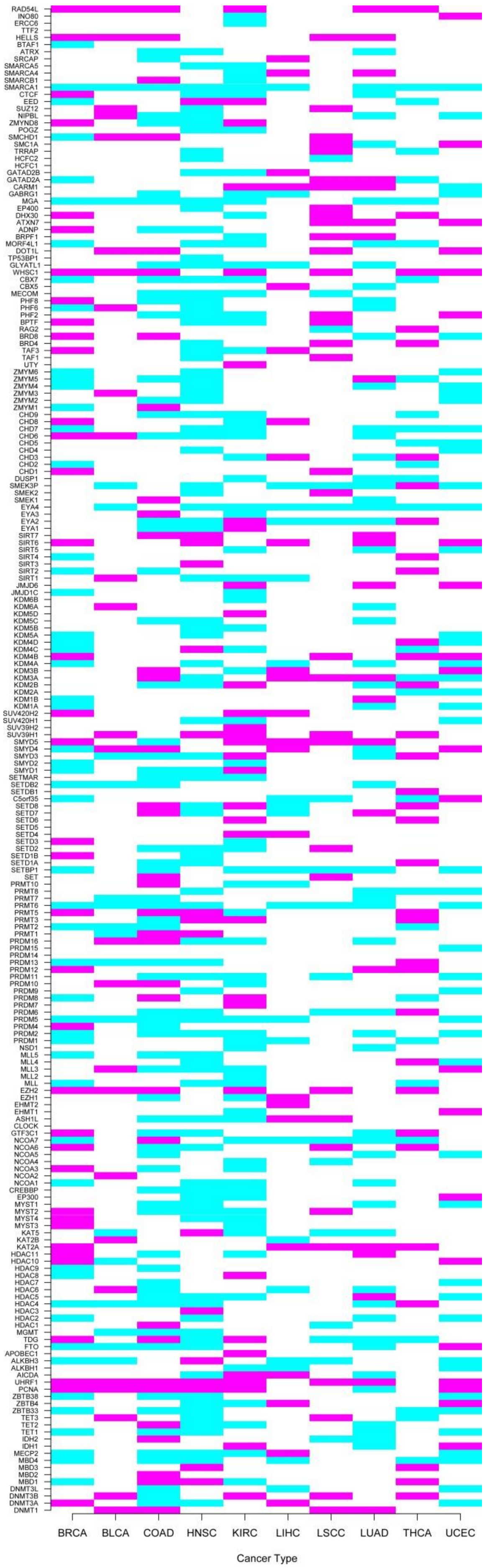
RAD54L
INO80
ERCC6
TTF2
HELLS
BTAF1
ATRX
SRCAP
SMARCA5
SMARCA4
SMARCB1
SMARCA1
CTCF
EED
SUZ12
NIPBL
ZMYND8
POGZ
SMCHD1
SMC1A
TRRAP
HCFC2
HCFC1
GATAD2B
GATAD2A
CARM1
GABRG1
MGA
EP400
DHX30
ATXN7
ADNP
BRPF1
MORF4L1
DOT1L
TP53BP1
GLYATL1
WHSC1
CBX7
CBX5
MECOM
PHF8
PHF6
PHF2
BPTF
RAG2
BRD8
BRD4
TAF3
TAF1
UTY
ZMYM6
ZMYM5
ZMYM4
ZMYM3
ZMYM2
ZMYM1
CHD9
CHD8
CHD7
CHD6
CHD5
CHD4
CHD3
CHD2
CHD1
DUSP1
SMEK3P
SMEK2
SMEK1
EYA4
EYA3
EYA2
EYA1
SIRT7
SIRT6
SIRT5
SIRT4
SIRT3
SIRT2
SIRT1
JMJD6
JMJD1C
KDM6B
KDM6A
KDM5D
KDM5C
KDM5B
KDM5A
KDM4D
KDM4C
KDM4B
KDM4A
KDM3B
KDM3A
KDM2B
KDM2A
KDM1B
KDM1A
SUV420H2
SUV420H1
SUV39H2
SUV39H1
SMYD5
SMYD4
SMYD3
SMYD2
SMYD1
SETMAR
SETDB2
SETDB1
C5orf35
SETD8
SETD7
SETD6
SETD5
SETD4
SETD3
SETD2
SETD1B
SETD1A
SETBP1
SET
PRMT10
PRMT8
PRMT7
PRMT6
PRMT5
PRMT3
PRMT2
PRMT1
PRDM16
PRDM15
PRDM14
PRDM13
PRDM12
PRDM11
PRDM10
PRDM9
PRDM8
PRDM7
PRDM6
PRDM5
PRDM4
PRDM2
PRDM1
NSD1
MLL5
MLL4
MLL3
MLL2
MLL
EZH2
EZH1
EHMT2
EHMT1
ASH1L
CLOCK
GTF3C1
NCOA7
NCOA6
NCOA5
NCOA4
NCOA3
NCOA2
NCOA1
CREBBP
EP300
MYST1
MYST2
MYST4
MYST3
KAT5
KAT2B
KAT2A
HDAC11
HDAC10
HDAC9
HDAC8
HDAC7
HDAC6
HDAC5
HDAC4
HDAC3
HDAC2
HDAC1
MGMT
TDG
FTO
APOBEC1
ALKBH3
ALKBH1
AICDA
UHRF1
PCNA
ZBTB38
ZBTB4
ZBTB33
TET3
TET2
TET1
IDH2
IDH1
MECP2
MBD4
MBD3
MBD2
MBD1
DNMT3L
DNMT3B
DNMT3A
DNMT1
BRCA
BLCA
COAD
HNSC
KIRC
LIHC
LSCC
LUAD
THCA
UCEC
Cancer Type

Figure.S4: Heatmap of Pearson Correlations between expression of EE genes and the HyperZ index, as assessed over cancer samples of a given cancer type. Color Codes: Magenta=significant positive correlation, White=no significant correlation, Cyan=significant negative correlation.

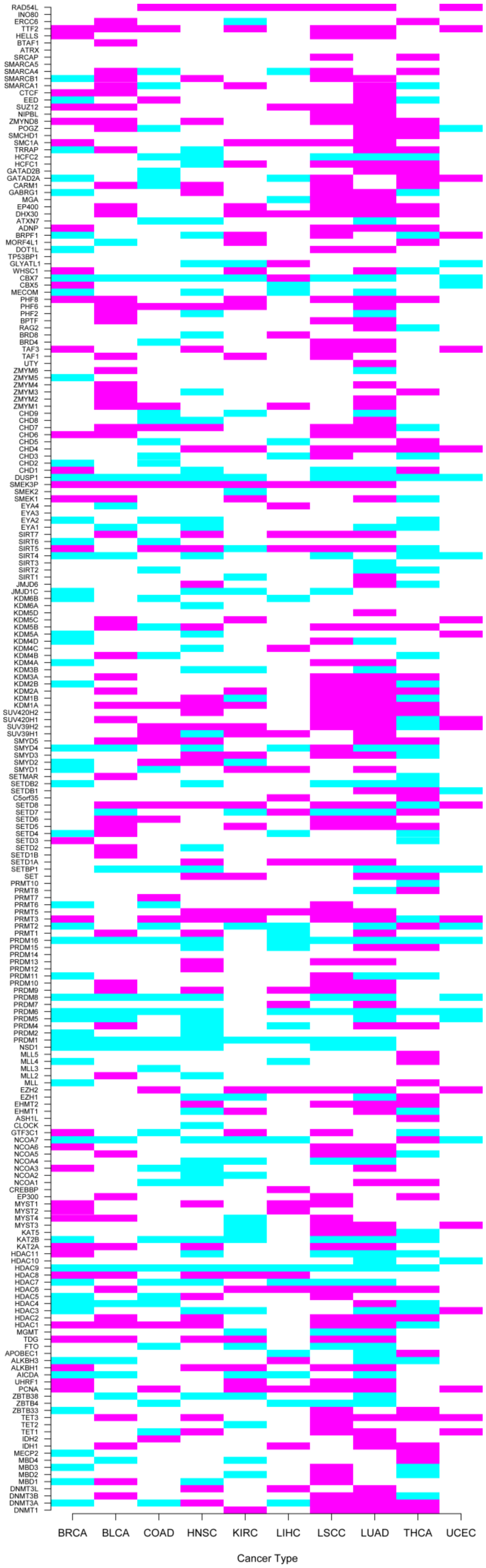
RAD54L
INO80
ERCC6
TTF2
HELLS
BTAF1
ATRX
SRCAP
SMARCA5
SMARCA4
SMARCB1
SMARCA1
CTCF
EED
SUZ12
NIPBL
ZMYND8
POGZ
SMCHD1
SMC1A
TRRAP
HCFC2
HCFC1
GATAD2B
GATAD2A
CARM1
GABRG1
MGA
EP400
DHX30
ATXN7
ADNP
BRPF1
MORF4L1
DOT1L
TP53BP1
GLYATL1
WHSC1
CBX7
CBX5
MECOM
PHF8
PHF6
PHF2
BPTF
RAG2
BRD8
BRD4
TAF3
TAF1
UTY
ZMYM6
ZMYM5
ZMYM4
ZMYM3
ZMYM2
ZMYM1
CHD9
CHD8
CHD7
CHD6
CHD5
CHD4
CHD3
CHD2
CHD1
DUSP1
SMEK3P
SMEK2
SMEK1
EYA4
EYA3
EYA2
EYA1
SIRT7
SIRT6
SIRT5
SIRT4
SIRT3
SIRT2
SIRT1
JMJD6
JMJD1C
KDM6B
KDM6A
KDM5D
KDM5C
KDM5B
KDM5A
KDM4D
KDM4C
KDM4B
KDM4A
KDM3B
KDM3A
KDM2B
KDM2A
KDM1B
KDM1A
SUV420H2
SUV420H1
SUV39H2
SUV39H1
SMYD5
SMYD4
SMYD3
SMYD2
SMYD1
SETMAR
SETDB2
SETDB1
C5orf35
SETD8
SETD7
SETD6
SETD5
SETD4
SETD3
SETD2
SETD1B
SETD1A
SETBP1
SET
PRMT10
PRMT8
PRMT7
PRMT6
PRMT5
PRMT3
PRMT2
PRMT1
PRDM16
PRDM15
PRDM14
PRDM13
PRDM12
PRDM11
PRDM10
PRDM9
PRDM8
PRDM7
PRDM6
PRDM5
PRDM4
PRDM2
PRDM1
NSD1
MLL5
MLL4
MLL3
MLL2
MLL
EZH2
EZH1
EHMT2
EHMT1
ASH1L
CLOCK
GTF3C1
NCOA7
NCOA6
NCOA5
NCOA4
NCOA3
NCOA2
NCOA1
CREBBP
EP300
MYST1
MYST2
MYST4
MYST3
KAT5
KAT2B
KAT2A
HDAC11
HDAC10
HDAC9
HDAC8
HDAC7
HDAC6
HDAC5
HDAC4
HDAC3
HDAC2
HDAC1
MGMT
TDG
FTO
APOBEC1
ALKBH3
ALKBH1
AICDA
UHRF1
PCNA
ZBTB38
ZBTB4
ZBTB33
TET3
TET2
TET1
IDH2
IDH1
MECP2
MBD4
MBD3
MBD2
MBD1
DNMT3L
DNMT3B
DNMT3A
DNMT1
BRCA
BLCA
COAD
HNSC
KIRC
LIHC
LSCC
LUAD
THCA
UCEC
Cancer Type

Figure.S5: Heatmap of Pearson Correlations between expression of EE genes and the HypoZ index, as assessed over cancer samples of a given cancer type. Color Codes: Magenta=significant positive correlation, White=no significant correlation, Cyan=significant negative correlation.

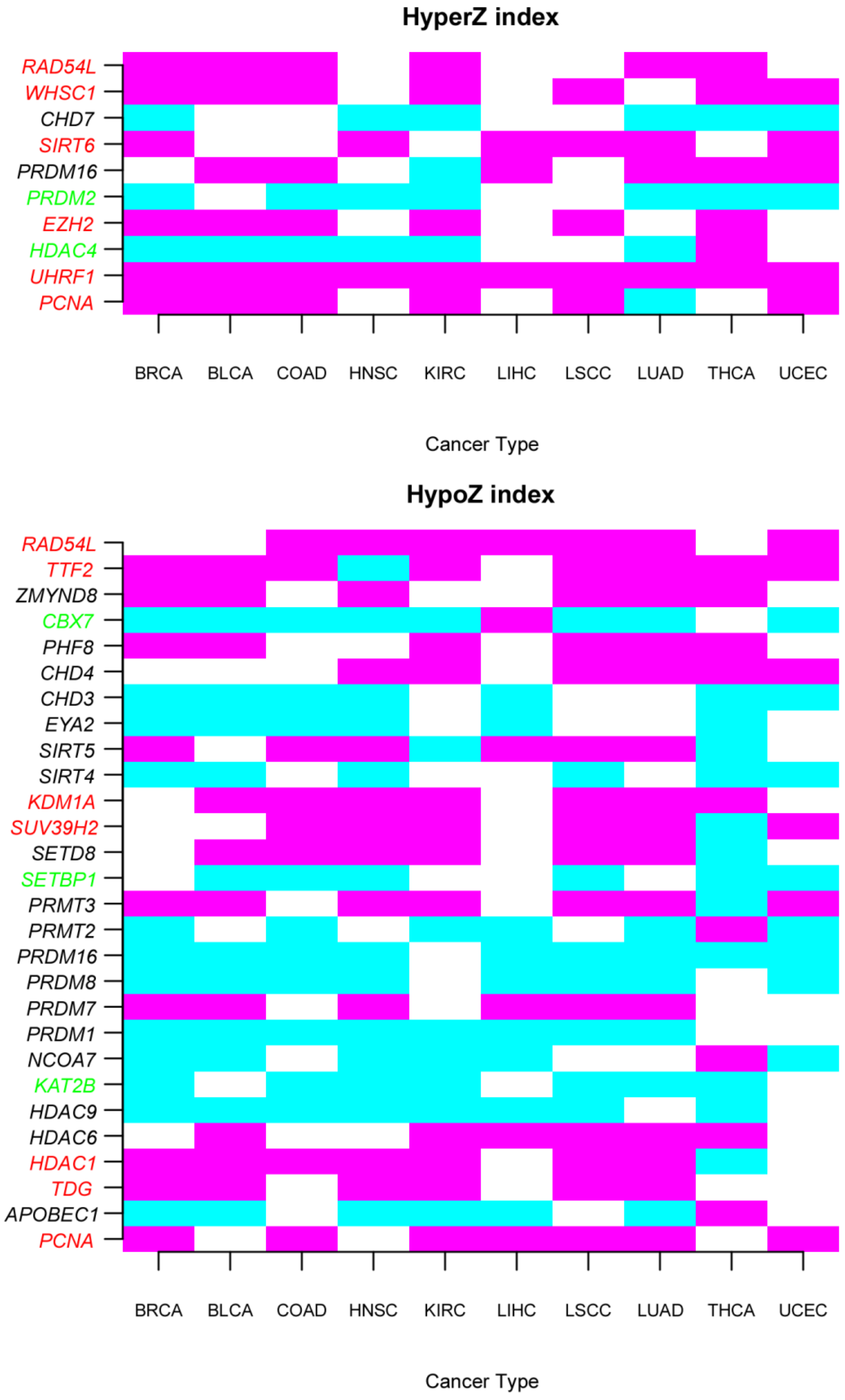

Figure.S6: Heatmap of Partial Correlations between mRNA expression of EE genes and the HyperZ and HypoZ index, respectively, as assessed over cancer samples of a given cancer type. Color Codes:

Magenta=significant positive partial correlation, White=no significant partial correlation, Cyan=significant negative partial correlation. Partial correlations were estimated from running multivariate regression models of the form *HyperZ/HypoZ ~ promoterDNAm(gene) + mRNAexpr.(gene) + error*.

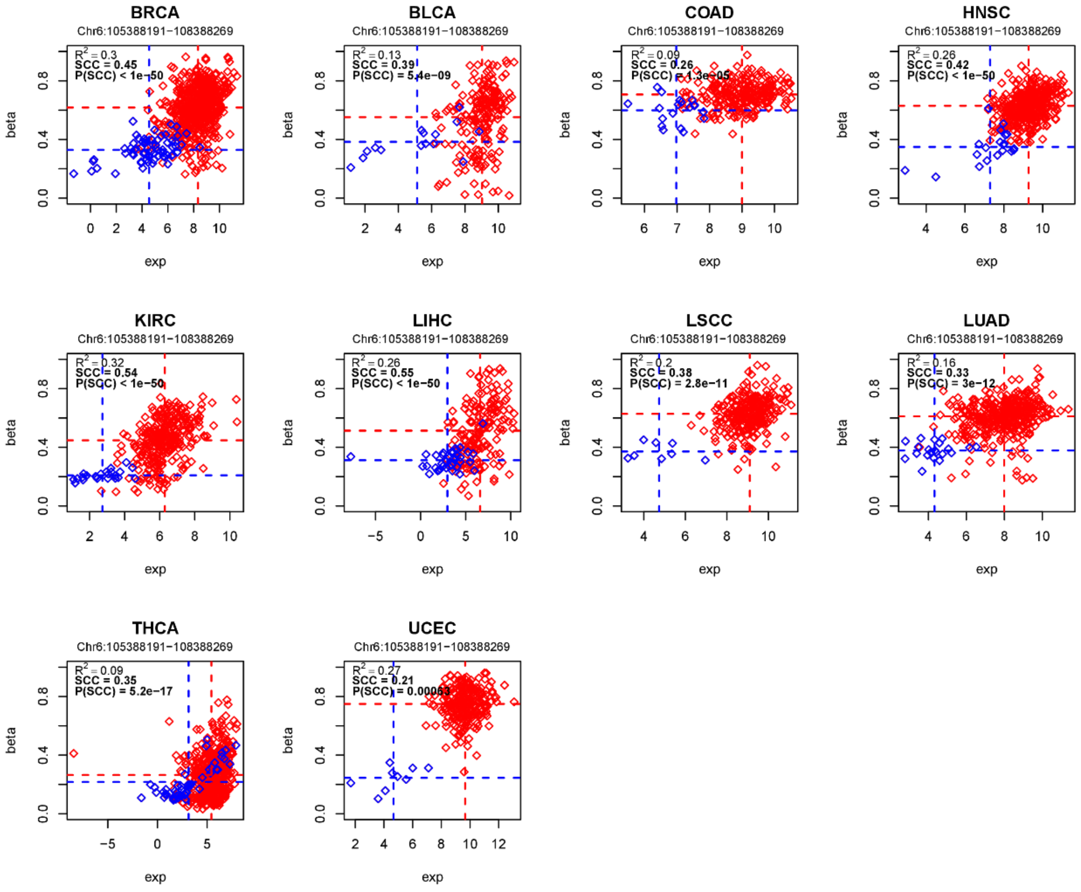

Figure.S7: Scatterplots of DNA methylation beta-value of a genomic CGI locus (as shown) with a relatively high HyperZ index across most cancer types (y-axis) against UHRF1 gene expression (log_2 level-3 RNA-Seq count, x-axis) for each cancer type as shown. Normal samples are shown in blue, cancer samples in red. Dashed horizontal and vertical lines represent the mean levels of DNAm and mRNA expression in the normal (blue) and cancers (red). R^2, Spearman Correlation Coefficient (SCC) and associated P-value are given in each plot. These numbers were calculated across cancer samples only.

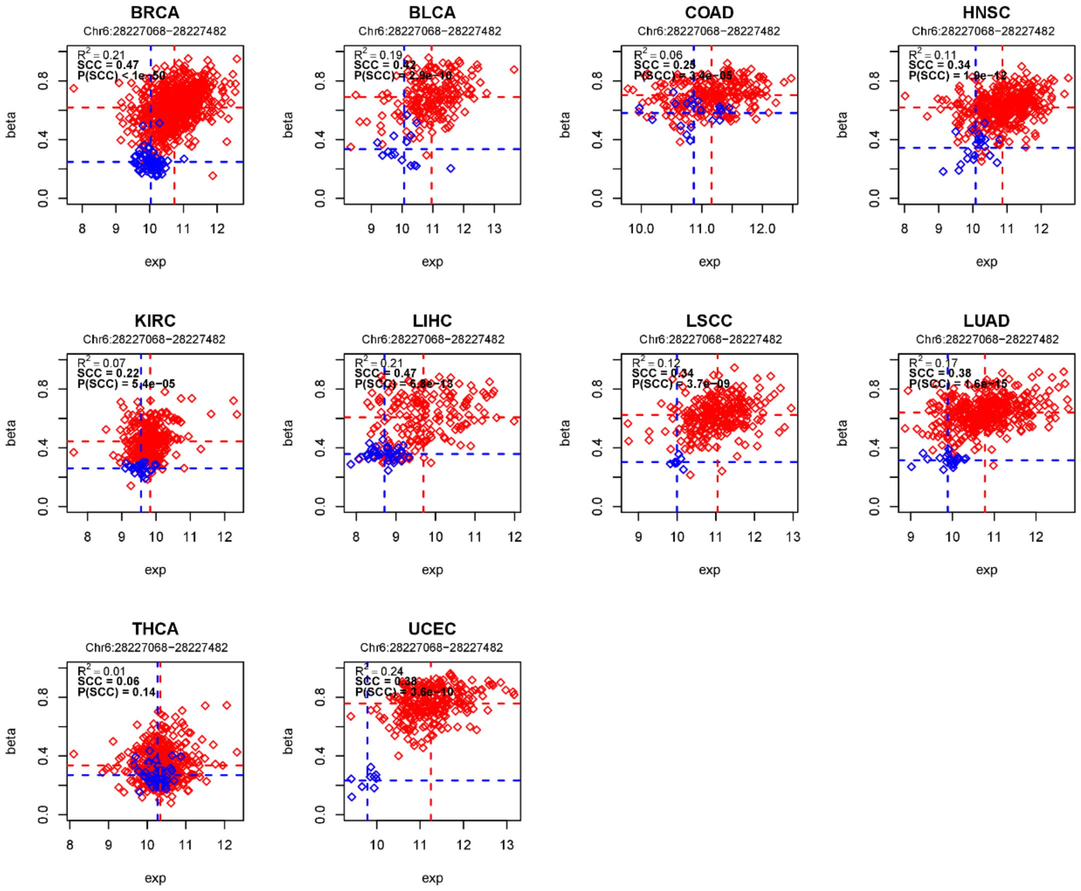

Figure.S8: Scatterplots of DNA methylation beta-value of a genomic CGI locus (as shown) with a relatively high HyperZ index across most cancer types (y-axis) against WHSC1 gene expression (log_2 level-3 RNA-Seq count, x-axis) for each cancer type as shown. Normal samples are shown in blue, cancer samples in red. Dashed horizontal and vertical lines represent the mean levels of DNAm and mRNA expression in the normal (blue) and cancers (red). R^2, Spearman Correlation Coefficient (SCC) and associated P-value are given in each plot. These numbers were calculated across cancer samples only.

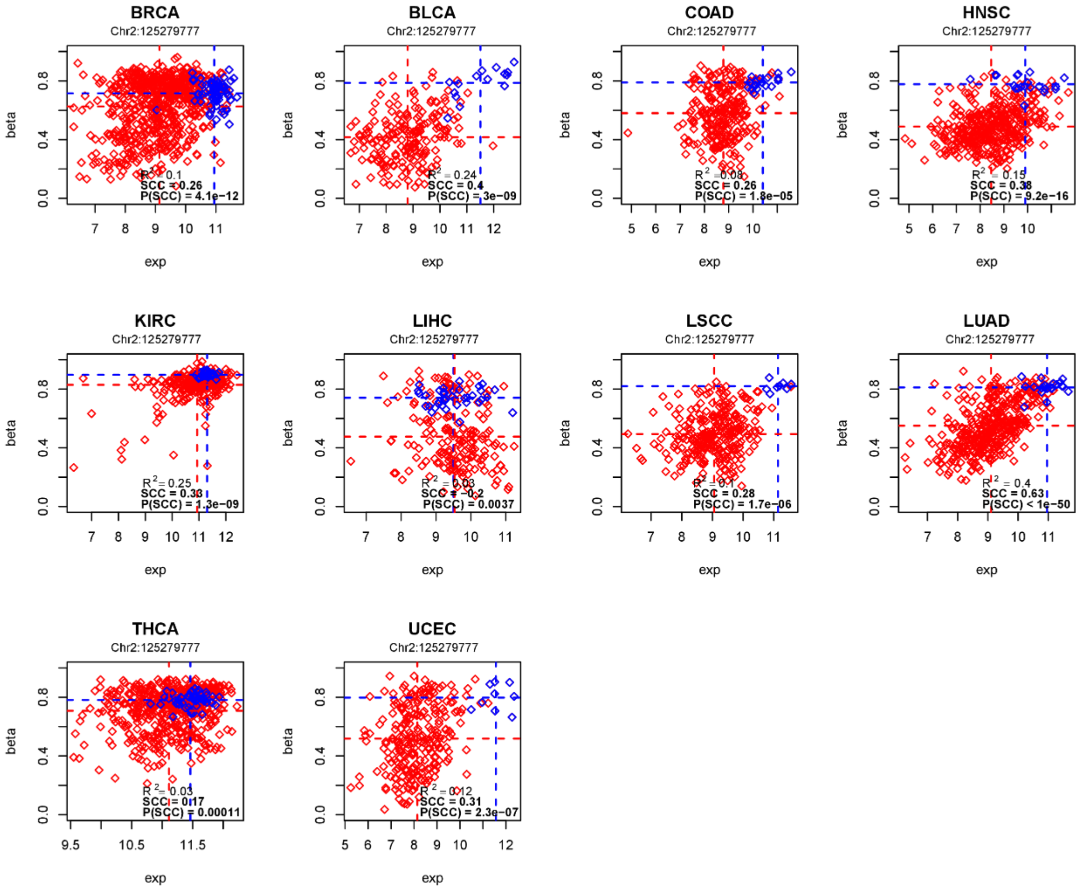

Figure.S9: Scatterplots of DNA methylation beta-value of a genomic open sea single probe locus (as shown) with a relatively high HypoZ index across most cancer types (y-axis) against CBX7 gene expression ($\log_2$ level-3 RNA-Seq count, x-axis) for each cancer type as shown. Normal samples are shown in blue, cancer samples in red. Dashed horizontal and vertical lines represent the mean levels of DNAm and mRNA expression in the normal (blue) and cancers (red). R^2, Spearman Correlation Coefficient (SCC) and associated P-value are given in each plot. These numbers were calculated across cancer samples only.

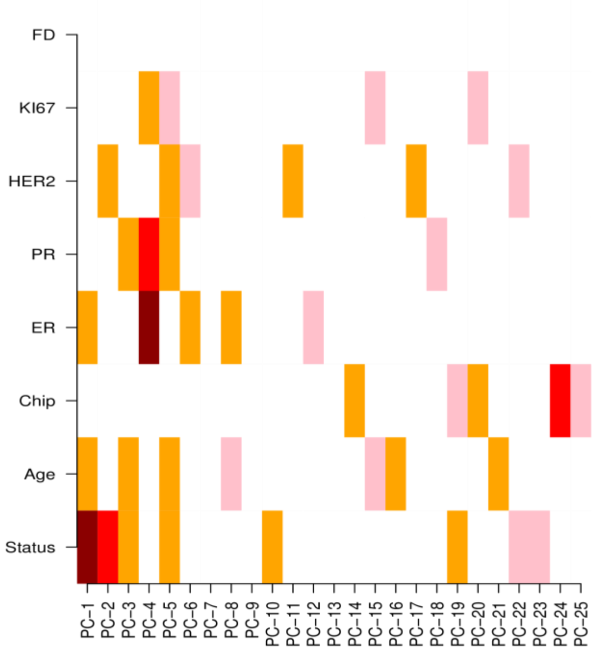

Figure.S10: Heatmap of associations between principal components (singular vectors) from a SVD and various factors for the Erlangen Breast cancer Illumina 450k DNA methylation study. The study included 50 normal samples from healthy women, 42 normal samples taken adjacent to a tumor, and over 200 breast cancers. The factors shown in the SVD heatmap include normal-cancer status (Status), Age, Beadchip, oestrogen receptor (ER), progesterone receptor (PR), HER2 status and KI67 status, as determined by immunohistochemistry. Also included is the comparison between normal-adjacent to normal-healthy, i.e. potential field defects (FD). Colors indicate statistical significance as follows: brown (P<1e-10), red (P<1e-5), orange (P<0.001), pink (P<0.05), white (not significant).

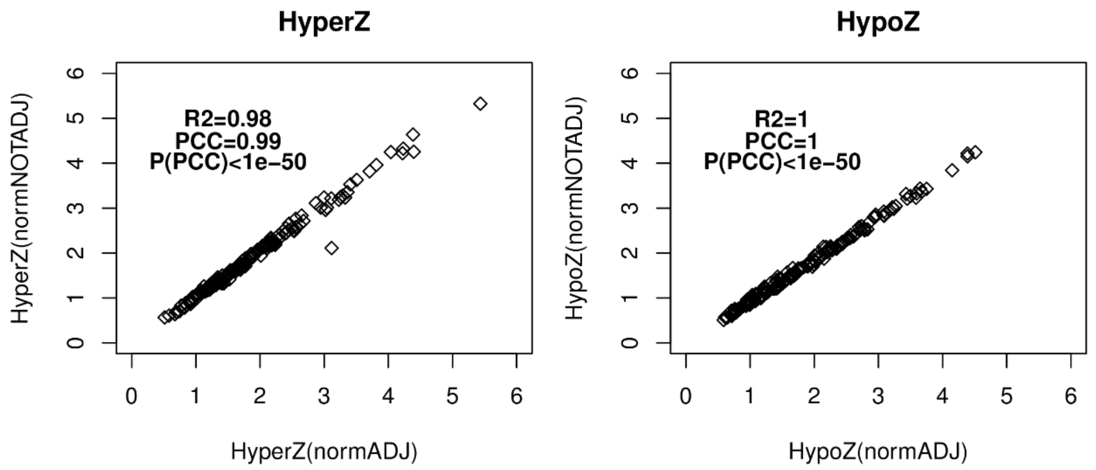

Figure.S11: Scatterplot of the HypoZ and HyperZ indices for the Erlangen Breast Cancer Illumina

450k study. In each case, y-axis labels the Z index of cancer samples as estimated relative to the 50 normal samples from healthy women, whereas the x-axis labels the corresponding Z-indices estimated relative to the 42 normal samples adjacent to tumors. R^2 values, Pearson Correlation Coefficient (PCC) and associated P-value are given.

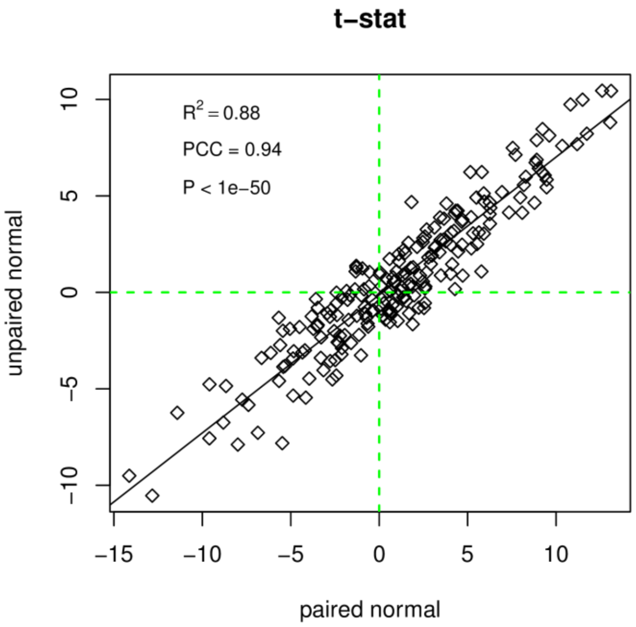

Figure.S12: Scatterplot of differential expression t-statistics of the 212 EE genes between normal colon and colon cancer samples of the TCGA, with the x-axis labeling the case where only paired normal samples were used (n=26) and y-axis labeling the case where only unpaired normal samples (n=12) were used. R^2 value, Pearson Correlation Coefficient (PCC) and associated P-value are given. Plot shows that the statistics of differential expression of the 212 EE genes would not alter significantly, had we used unpaired normal samples as reference.

SUPPLEMENTARY TABLES

Gene Symbol	Gene Family	Group	Functional class
DNMT1	DNA (cytosine-5-)-methyltransferase 1 (DNMT)	DNA modification	DNA methylation maintain
DNMT3A	DNA (cytosine-5-)-methyltransferase 2	DNA modification	De novo methylation

	(DNMT)		
DNMT3B	DNA (cytosine-5-)-methyltransferase 3 (DNMT)	DNA modification	De novo methylation
DNMT3L	DNA (cytosine-5-)-methyltransferase 4 (DNMT)	DNA modification	De novo methylation
MBD1	Methyl-CpG-Binding Protein (MBD)	DNA modification	DNA methylation reader
MBD2	Methyl-CpG-Binding Protein (MBD)	DNA modification	DNA methylation reader
MBD3	Methyl-CpG-Binding Protein (MBD)	DNA modification	DNA methylation reader
MBD4	Methyl-CpG-Binding Protein (MBD)	DNA modification	DNA methylation reader
MECP2	Methyl-CpG-Binding Protein (MBD)	DNA modification	DNA methylation reader
IDH1	Isocitrate dehydrogenase 1 (IDH)	DNA modification	DNA methylation editor/eraser
IDH2	Isocitrate dehydrogenase 2 (IDH)	DNA modification	DNA methylation editor/eraser
TET1	Ten-eleven translocation methylcytosine dioxygenase (TET)	DNA modification	DNA methylation editor/eraser
TET2	Ten-eleven translocation methylcytosine dioxygenase (TET)	DNA modification	DNA methylation editor/eraser
TET3	Ten-eleven translocation methylcytosine dioxygenase (TET)	DNA modification	DNA methylation editor/eraser
ZBTB33	Zinc finger and BTB domain containing (ZBTB)	DNA modification	DNA methylation reader
ZBTB4	Zinc finger and BTB domain containing (ZBTB)	DNA modification	DNA methylation reader
ZBTB38	Zinc finger and BTB domain containing (ZBTB)	DNA modification	DNA methylation reader
PCNA	proliferating cell nuclear antigen	DNA modification	DNA methylation reader
UHRF1	ubiquitin-like with PHD and ring finger domains 1	DNA modification	DNA methylation reader
AICDA	cytidine deaminase family	DNA modification	DNA methylation editor/eraser
ALKBH1	alkylation repair homolog	DNA modification	DNA methylation editor/eraser
ALKBH3	alkylation repair homolog	DNA modification	DNA methylation editor/eraser
APOBEC1	cytidine deaminase enzyme	DNA modification	DNA methylation editor/eraser
FTO	fat mass and obesity associated	DNA modification	DNA methylation editor/eraser
TDG	TDG/mug DNA glycosylase family	DNA modification	DNA methylation editor/eraser
MGMT	O-6-methylguanine-DNA methyltransferase	DNA modification	DNA methylation editor/eraser
HDAC1	Histone deacetylase (HDAC)	Histone Modification	K27 eraser
HDAC2	Histone deacetylase (HDAC)	Histone Modification	K27 eraser
HDAC3	Histone deacetylase (HDAC)	Histone Modification	K27 eraser
HDAC4	Histone deacetylase (HDAC)	Histone Modification	K27 eraser
HDAC5	Histone deacetylase (HDAC)	Histone Modification	K27 eraser
HDAC6	Histone deacetylase (HDAC)	Histone Modification	K27 eraser
HDAC7	Histone deacetylase (HDAC)	Histone Modification	K27 eraser
HDAC8	Histone deacetylase (HDAC)	Histone Modification	K27 eraser
HDAC9	Histone deacetylase (HDAC)	Histone Modification	K27 eraser

HDAC10	Histone deacetylase (HDAC)	Histone Modification	K27 eraser
HDAC11	Histone deacetylase (HDAC)	Histone Modification	K27 eraser
KAT2A	Histone acetyltransferases (HAT)	Histone Modification	acetylation writer
KAT2B	Histone acetyltransferases (HAT)	Histone Modification	acetylation writer
KAT5	Histone acetyltransferases (HAT)	Histone Modification	acetylation writer
MYST3	Histone acetyltransferases (HAT) KAT6A	Histone Modification	acetylation writer
MYST4	Histone acetyltransferases (HAT) KAT6B	Histone Modification	acetylation writer
MYST2	Histone acetyltransferases (HAT) KAT7	Histone Modification	acetylation writer
MYST1	Histone acetyltransferases (HAT) KAT8	Histone Modification	acetylation writer
EP300	E1A binding protein p300	Histone Modification	Histone acetyltransferases
CREBBP	CREB binding protein	Histone Modification	Histone acetyltransferases
NCOA1	p160/steroid receptor coactivator (SRC) family	Histone Modification	Histone acetyltransferases
NCOA2	p161/steroid receptor coactivator (SRC) family	Histone Modification	Histone acetyltransferases
NCOA3	p162/steroid receptor coactivator (SRC) family	Histone Modification	Histone acetyltransferases
NCOA4	p163/steroid receptor coactivator (SRC) family	Histone Modification	Histone acetyltransferases
NCOA5	p164/steroid receptor coactivator (SRC) family	Histone Modification	Histone acetyltransferases
NCOA6	p165/steroid receptor coactivator (SRC) family	Histone Modification	Histone acetyltransferases
NCOA7	p166/steroid receptor coactivator (SRC) family	Histone Modification	Histone acetyltransferases
GTF3C1	general transcription factor IIIC, polypeptide	Histone Modification	Histone acetyltransferases
CLOCK	clock circadian regulator	Histone Modification	Histone acetyltransferases
ASH1L	ash1 (absent, small, or homeotic)-like	Histone Modification	Histone methyltransferases (HMT)
EHMT1	euchromatic histone-lysine N-methyltransferase	Histone Modification	Histone methyltransferases (HMT)
EHMT2	euchromatic histone-lysine N-methyltransferase	Histone Modification	Histone methyltransferases (HMT)
EZH1	enhancer of zeste 1 polycomb repressive complex 2 subunit	Histone Modification	Histone methyltransferases (HMT)
EZH2	enhancer of zeste 1 polycomb repressive complex 3 subunit	Histone Modification	Histone methyltransferases (HMT)
MLL	lysine (K)-specific methyltransferase (KMT2A)	Histone Modification	Histone methyltransferases (HMT)
MLL2	lysine (K)-specific methyltransferase (KMT2D)	Histone Modification	Histone methyltransferases (HMT)
MLL3	lysine (K)-specific methyltransferase (KMT2C)	Histone Modification	Histone methyltransferases (HMT)

MLL4	lysine (K)-specific methyltransferase (KMT2B)	Histone Modification	Histone methyltransferases (HMT)
MLL5	lysine (K)-specific methyltransferase (KMT2E)	Histone Modification	Histone methyltransferases (HMT)
NSD1	nuclear receptor binding SET domain protein 1	Histone Modification	Histone methyltransferases (HMT)
PRDM1	PR domain containing	Histone Modification	Histone methyltransferases
PRDM2	PR domain containing	Histone Modification	Histone methyltransferases
PRDM4	PR domain containing	Histone Modification	Histone methyltransferases
PRDM5	PR domain containing	Histone Modification	Histone methyltransferases
PRDM6	PR domain containing	Histone Modification	Histone methyltransferases
PRDM7	PR domain containing	Histone Modification	Histone methyltransferases
PRDM8	PR domain containing	Histone Modification	Histone methyltransferases
PRDM9	PR domain containing	Histone Modification	Histone methyltransferases
PRDM10	PR domain containing	Histone Modification	Histone methyltransferases
PRDM11	PR domain containing	Histone Modification	Histone methyltransferases
PRDM12	PR domain containing	Histone Modification	Histone methyltransferases
PRDM13	PR domain containing	Histone Modification	Histone methyltransferases
PRDM14	PR domain containing	Histone Modification	Histone methyltransferases
PRDM15	PR domain containing	Histone Modification	Histone methyltransferases
PRDM16	PR domain containing	Histone Modification	Histone methyltransferases
PRMT1	protein arginine N-methyltransferase (PRMT)	Histone Modification	Histone methyltransferases
PRMT2	protein arginine N-methyltransferase (PRMT)	Histone Modification	Histone methyltransferases
PRMT3	protein arginine N-methyltransferase (PRMT)	Histone Modification	Histone methyltransferases
PRMT5	protein arginine N-methyltransferase (PRMT)	Histone Modification	Histone methyltransferases
PRMT6	protein arginine N-methyltransferase (PRMT)	Histone Modification	Histone methyltransferases
PRMT7	protein arginine N-methyltransferase (PRMT)	Histone Modification	Histone methyltransferases
PRMT8	protein arginine N-methyltransferase (PRMT)	Histone Modification	Histone methyltransferases
PRMT10	protein arginine N-methyltransferase (PRMT)	Histone Modification	Histone methyltransferases
SET	SET nuclear proto-oncogene	Histone Modification	histone acetylases (HAT)
SETBP1	SET binding protein 1	Histone Modification	Histone methyltransferases (HMT)
SETD1A	SET domain containing	Histone Modification	Histone methyltransferases (HMT)
SETD1B	SET domain containing	Histone Modification	Histone methyltransferases (HMT)

SETD2	SET domain containing	Histone Modification	Histone methyltransferases (HMT)
SETD3	SET domain containing	Histone Modification	Histone methyltransferases (HMT)
SETD4	SET domain containing	Histone Modification	Histone methyltransferases (HMT)
SETD5	SET domain containing	Histone Modification	Histone methyltransferases (HMT)
SETD6	SET domain containing	Histone Modification	Histone methyltransferases (HMT)
SETD7	SET domain containing	Histone Modification	Histone methyltransferases (HMT)
SETD8	SET domain containing	Histone Modification	Histone methyltransferases (HMT)
C5orf35	SET domain containing (SETD9)	Histone Modification	Histone methyltransferases (HMT)
SETDB1	SET domain containing	Histone Modification	Histone methyltransferases (HMT)
SETDB2	SET domain containing	Histone Modification	Histone methyltransferases (HMT)
SETMAR	SET domain containing	Histone Modification	Histone methyltransferases (HMT)
SMYD1	SET and MYND domain containing	Histone Modification	Histone methyltransferases (HMT)
SMYD2	SET and MYND domain containing	Histone Modification	Histone methyltransferases (HMT)
SMYD3	SET and MYND domain containing	Histone Modification	Histone methyltransferases (HMT)
SMYD4	SET and MYND domain containing	Histone Modification	Histone methyltransferases (HMT)
SMYD5	SET and MYND domain containing	Histone Modification	Histone methyltransferases (HMT)
SUV39H1	suppressor of variegation 3-9	Histone Modification	Histone methyltransferases (HMT)
SUV39H2	suppressor of variegation 3-9	Histone Modification	Histone methyltransferases (HMT)
SUV420H1	suppressor of variegation 4-20 homolog	Histone Modification	Histone methyltransferases (HMT)
SUV420H2	suppressor of variegation 4-20 homolog	Histone Modification	Histone methyltransferases (HMT)
KDM1A	lysine (K)-specific demethylase (KDM)	Histone Modification	Histone demethylase (HDM)
KDM1B	lysine (K)-specific demethylase (KDM)	Histone Modification	Histone demethylase (HDM)
KDM2A	lysine (K)-specific demethylase (KDM)	Histone Modification	Histone demethylase (HDM)
KDM2B	lysine (K)-specific demethylase (KDM)	Histone Modification	Histone demethylase (HDM)

KDM3A	lysine (K)-specific demethylase (KDM)	Histone Modification	Histone demethylase (HDM)
KDM3B	lysine (K)-specific demethylase (KDM)	Histone Modification	Histone demethylase (HDM)
KDM4A	lysine (K)-specific demethylase (KDM)	Histone Modification	Histone demethylase (HDM)
KDM4B	lysine (K)-specific demethylase (KDM)	Histone Modification	Histone demethylase (HDM)
KDM4C	lysine (K)-specific demethylase (KDM)	Histone Modification	Histone demethylase (HDM)
KDM4D	lysine (K)-specific demethylase (KDM)	Histone Modification	Histone demethylase (HDM)
KDM5A	lysine (K)-specific demethylase (KDM)	Histone Modification	Histone demethylase (HDM)
KDM5B	lysine (K)-specific demethylase (KDM)	Histone Modification	Histone demethylase (HDM)
KDM5C	lysine (K)-specific demethylase (KDM)	Histone Modification	Histone demethylase (HDM)
KDM5D	lysine (K)-specific demethylase (KDM)	Histone Modification	Histone demethylase (HDM)
KDM6A	lysine (K)-specific demethylase (KDM)	Histone Modification	Histone demethylase (HDM)
KDM6B	lysine (K)-specific demethylase (KDM)	Histone Modification	Histone demethylase (HDM)
JMJD1C	jumonji domain containing	Histone Modification	protein hydroxylases or histone demethylases
JMJD6	jumonji domain containing	Histone Modification	protein hydroxylases or histone demethylases
SIRT1	sirtuin family	Histone Modification	acetylation editor
SIRT2	sirtuin family	Histone Modification	acetylation editor
SIRT3	sirtuin family	Histone Modification	acetylation editor
SIRT4	sirtuin family	Histone Modification	acetylation editor
SIRT5	sirtuin family	Histone Modification	acetylation editor
SIRT6	sirtuin family	Histone Modification	acetylation editor
SIRT7	sirtuin family	Histone Modification	acetylation editor
EYA1	eyes absent (EYA) family	Histone Modification	Phosphorylation editor
EYA2	eyes absent (EYA) family	Histone Modification	Phosphorylation editor
EYA3	eyes absent (EYA) family	Histone Modification	Phosphorylation editor
EYA4	eyes absent (EYA) family	Histone Modification	Phosphorylation editor
SMEK1	SMEK homolog	Histone Modification	Phosphorylation editor
SMEK2	SMEK homolog	Histone Modification	Phosphorylation editor
SMEK3P	SMEK homolog	Histone Modification	Phosphorylation editor
DUSP1	dual specificity phosphatase 1	Histone Modification	Phosphorylation editor
CHD1	chromodomain helicase DNA binding protein (CHD)	Histone Modification	Acetylation, methylation and phosphorylation Reader
CHD2	chromodomain helicase DNA binding protein (CHD)	Histone Modification	Acetylation, methylation and phosphorylation Reader
CHD3	chromodomain helicase DNA binding protein (CHD)	Histone Modification	Acetylation, methylation and phosphorylation Reader
CHD4	chromodomain helicase DNA binding protein (CHD)	Histone Modification	Acetylation, methylation and phosphorylation Reader
CHD5	chromodomain helicase DNA binding protein (CHD)	Histone Modification	Acetylation, methylation and phosphorylation Reader
CHD6	chromodomain helicase DNA binding protein (CHD)	Histone Modification	Acetylation, methylation and phosphorylation Reader
CHD7	chromodomain helicase DNA binding	Histone Modification	Acetylation, methylation and

	protein (CHD)		phosphorylation Reader
CHD8	chromodomain helicase DNA binding protein (CHD)	Histone Modification	Acetylation, methylation and phosphorylation Reader
CHD9	chromodomain helicase DNA binding protein (CHD)	Histone Modification	Acetylation, methylation and phosphorylation Reader
ZMYM1	zinc finger, MYM-type	Histone Modification	Acetylation, methylation and phosphorylation Reader
ZMYM2	zinc finger, MYM-type	Histone Modification	Acetylation, methylation and phosphorylation Reader
ZMYM3	zinc finger, MYM-type	Histone Modification	Acetylation, methylation and phosphorylation Reader
ZMYM4	zinc finger, MYM-type	Histone Modification	Acetylation, methylation and phosphorylation Reader
ZMYM5	zinc finger, MYM-type	Histone Modification	Acetylation, methylation and phosphorylation Reader
ZMYM6	zinc finger, MYM-type	Histone Modification	Acetylation, methylation and phosphorylation Reader
UTY	ubiquitously transcribed tetratricopeptide repeat containing, Y-linked	Histone Modification	Histone demethylase (HDM)
TAF1	TATA box binding protein (TBP)-associated factor	Histone Modification	Acetylation, methylation and phosphorylation
TAF3	TATA box binding protein (TBP)-associated factor	Histone Modification	K4 reader
BRD4	bromodomain containing	Histone Modification	K27 reader
BRD8	bromodomain containing	Histone Modification	K27 reader
RAG2	recombination activating gene 2	Histone Modification	K4 reader
BPTF	bromodomain PHD finger transcription factor	Histone Modification	K4 reader
PHF2	PHD finger protein	Histone Modification	K4 reader
PHF6	PHD finger protein	Histone Modification	K4 reader
PHF8	PHD finger protein	Histone Modification	K4 reader
MECOM	MDS1 and EVI1 complex locus	Histone Modification	K9 writer
CBX5	chromobox homolog	Histone Modification	K9 reader
CBX7	chromobox homolog	Histone Modification	K36 reader
WHSC1	Wolf-Hirschhorn syndrome candidate 1	Histone Modification	K36 writer
GLYATL1	glycine-N-acyltransferase-like 1 (GNAT)	Histone Modification	K27 writer
TP53BP1	tumor protein p53 binding protein 1	Histone Modification	K79 reader
DOT1L	DOT1-like histone H3K79 methyltransferase	Histone Modification	K79 writer
MORF4L1	mortality factor 4 like 1	Histone Modification	K36 reader
BRPF1	bromodomain and PHD finger containing, 1	Histone Modification	K36 reader
ADNP	activity-dependent neuroprotector homeobox	Histone Modification	Acetylation, methylation and phosphorylation Reader

ATXN7	ataxin 7	Histone Modification	Acetylation, methylation and phosphorylation Reader
DHX30	DEAH (Asp-Glu-Ala-His) box helicase 30	Histone Modification	Acetylation, methylation and phosphorylation Reader
EP400	E1A binding protein	Histone Modification	Acetylation, methylation and phosphorylation Reader
MGA	MAX dimerization protein	Histone Modification	Acetylation, methylation and phosphorylation Reader
GABRG1	gamma-aminobutyric acid (GABA) A receptor, gamma 1	Histone Modification	Acetylation, methylation and phosphorylation Reader
CARM1	coactivator-associated arginine methyltransferase 1	Histone Modification	Acetylation, methylation and phosphorylation Reader
GATAD2A	GATA zinc finger domain containing	Histone Modification	Acetylation, methylation and phosphorylation Reader
GATAD2B	GATA zinc finger domain containing	Histone Modification	Acetylation, methylation and phosphorylation Reader
HCFC1	host cell factor family	Histone Modification	Acetylation, methylation and phosphorylation Reader
HCFC2	host cell factor family	Histone Modification	Acetylation, methylation and phosphorylation Reader
TRRAP	phosphoinositide 3-kinase-related kinases (PIKK) family	Histone Modification	Acetylation, methylation and phosphorylation Reader
SMC1A	structural maintenance of chromosomes 1A	Histone Modification	Acetylation, methylation and phosphorylation Reader
SMCHD1	structural maintenance of chromosomes flexible hinge domain containing 1	Histone Modification	Acetylation, methylation and phosphorylation Reader
POGZ	pogo transposable element with ZNF domain	Histone Modification	Acetylation, methylation and phosphorylation Reader
ZMYND8	zinc finger, MYND-type containing 8	Histone Modification	Acetylation, methylation and phosphorylation Reader
NIPBL	Nipped-B homolog	Histone Modification	Acetylation, methylation and phosphorylation Reader
SUZ12	Polycomb group protein	Histone Modification	chromatin silencing
EED	Polycomb group protein	Histone Modification	histone deacetylation
CTCF	CTCF gene family	Histone Modification	histone acetyltransferase or deacetylase
SMARCA1	SWI/SNF family	Nucleosome Positioning and Remodeling	Chromatin remodelling helicase
SMARCB1	SWI/SNF family	Nucleosome Positioning and Remodeling	Chromatin remodelling helicase
SMARCA4	SWI/SNF family	Nucleosome Positioning and Remodeling	Chromatin remodelling helicase
SMARCA5	SWI/SNF family	Nucleosome Positioning and Remodeling	Chromatin remodelling helicase

SRCAP	Snf2-related CREBBP activator protein	Nucleosome Positioning and Remodeling	Chromatin remodelling helicase
ATRX	alpha thalassemia/mental retardation syndrome X-linked	Nucleosome Positioning and Remodeling	Chromatin remodelling helicase
BTAF1	BTAF1 RNA polymerase II, B-TFIID transcription factor-associated	Nucleosome Positioning and Remodeling	Chromatin remodelling helicase
HELLS	helicase, lymphoid-specific	Nucleosome Positioning and Remodeling	Chromatin remodelling helicase
TTF2	SWI2/SNF2 family	Nucleosome Positioning and Remodeling	Chromatin remodelling helicase
ERCC6	excision repair cross-complementation group 6	Nucleosome Positioning and Remodeling	Chromatin remodelling helicase
INO80	INO80 complex subunit	Nucleosome Positioning and Remodeling	Chromatin remodelling helicase
RAD54L	DEAD-like helicase superfamily	Nucleosome Positioning and Remodeling	Chromatin remodelling helicase

Table.S1: The full list of 212 Epigenetic Enzyme genes, loosely defined as genes with a role in regulating/modulating any epigenetic mark. We provide the gene symbol, the gene description, its functional role and gene family it belongs to.

Cancer Type	#N(DNAm)	#C(DNAm)	#N(mRNA)	#C(mRNA)	#MN(DNAm)	#MC(mRNA)
**BRCA**	81	652	97	1008	68	648
**BLCA**	19	201	17	323	15	195
**COAD**	38	272	41	270	19	254
**HNSC**	45	405	42	475	20	396
**KIRC**	160	299	72	515	24	297
**LIHC**	47	176	50	349	38	173
**LSCC**	41	275	45	473	8	275
**LUAD**	32	399	58	471	21	392
**THCA**	53	489	56	495	48	487
**UCEC**	34	374	10	364	9	253

Table.S2: Numbers of normal (#N) and cancer (#C) tissue samples for each tissue type, and for each data type (DNAm=Illumina 450k DNA methylation, mRNA=RNA-SeqV3). Numbers of normals (#MN) and cancers (#MC) with matched DNAm and mRNA data are also given.

	BLCA	BRCA	COAD	HNSC	KIRC	LIHC	LSCC	LUAD	THCA	UCEC	Mean
ALL	20231	20248	20037	20253	20247	20164	20243	20190	20161	20358	20213.2
nDEG	10392	15238	14320	12249	15421	13111	15154	14342	13822	10116	13416.5
nDN	5097	8421	7934	6086	7814	5748	7697	6673	8593	5308	6937.1
nUP	5295	6817	6386	6163	7607	7363	7457	7669	5229	4808	6479.4
pDEG	0.51	0.75	0.71	0.6	0.76	0.65	0.75	0.71	0.69	0.5	0.66
pDN	0.25	0.42	0.4	0.3	0.39	0.29	0.38	0.33	0.43	0.26	0.34
pUP	0.26	0.34	0.32	0.3	0.38	0.37	0.37	0.38	0.26	0.24	0.32

Table.S3: For each TCGA cancer data set, we list the total number of genes which underwent Differential Expression analysis (ALL), the number of differentially expressed genes passing a P-value threshold of 0.05 (nDEG), the number of these which are downregulated (nDN) or upregulated (nUP) in cancer, and the corresponding probabilities of differential expression (pDEG), downregulation (pDN) and upregulation (pUP). We also provide the average values over all 10 cancer types in the last column.

	nEE (Observed)	P (k≥8)	nEE (212*P) (Expected)	SD	Binomial P-value
UP	35	0.0026	0.54	0.74	$<10^{-50}$
DN	27	0.0042	0.89	0.94	$<10^{-30}$

Table.S4: Table summarizing the analytical model result for estimating the overall statistical significance of observing 35 (27) significantly and consistently upregulated (downregulated) EE genes across at least 8 of the 10 cancer types. UP=upregulated, DN=downregulated. Columns label the observed numbers of EE genes (nEE), the null probability of observing a random gene (i.e. one of the ~20,000 genes) to be significantly and consistently differentially expressed across at least 8 of the 10 cancer types (P, k≥8), the number of EE genes expected under the Binomial test (there were 212 EE genes), the standard deviation of the Binomial count (SD) and the corresponding Binomial test P-value. We can see that for a random set of 212 genes, that the expected numbers of significantly and consistently differentially expressed genes across at least 8 of the 10 cancer types, are very low, approximately 0 to 1 genes in the case of upregulation, and only 0 to 2 genes in the case of downregulation.

	BRCA	BLCA	COAD	HNSC	KIRC	LIHC	LSCC	LUAD	THCA	UCEC	Mean

ALL	20219	20176	20008	20216	20214	20062	20222	20154	20154	20354	20177.9
Positive-HyperZ	3420 (0.17)	1340 (0.07)	2888 (0.14)	2048 (0.1)	3855 (0.19)	1259 (0.06)	1717 (0.08)	2462 (0.12)	2895 (0.14)	1616 (0.08)	2350 (0.12)
Negative-HyperZ	6799 (0.34)	2454 (0.12)	6053 (0.3)	7761 (0.38)	4590 (0.23)	3810 (0.19)	3565 (0.18)	5017 (0.25)	4602 (0.23)	5446 (0.27)	5009.7 (0.25)
Positive-HypoZ	3176 (0.16)	2528 (0.13)	1708 (0.09)	4787 (0.24)	3028 (0.15)	3296 (0.16)	3940 (0.19)	5022 (0.25)	4072 (0.2)	1213 (0.06)	3277 (0.16)
Negative-HypoZ	7470 (0.37)	4608 (0.23)	4995 (0.25)	5297 (0.26)	4610 (0.23)	4200 (0.21)	4715 (0.23)	6657 (0.33)	5914 (0.29)	2876 (0.14)	5134.2 (0.25)

Table.S5: For each TCGA cancer data set, we list the total number of genes (ALL) which underwent correlation analysis between mRNA expression and the HyperZ or HypoZ indices, the number of genes correlating significantly (i.e. with a correlation $P < 0.05$) and positively with HyperZ index, the number of genes correlating significantly and negatively with HyperZ, the number of genes correlating significantly and positively with the HypoZ index, and finally, the number of genes correlating significantly and negatively with the HypoZ index. In brackets, we give the corresponding fractions/probabilities. The last columns labels the mean over all cancer-types.

	nEE (Observed)	P (k≥6)	nEE (212*P) (Expected)	SD	Binomial P-value
positive-HyperZ (uu)	5	0.0004	0.08	0.29	$<10^{-9}$
negative-HyperZ (du)	11	0.02	4.24	2.04	0.001
positive-HypoZ (ud)	18	0.002	0.42	0.65	$<10^{-24}$
negative-hypoZ (dd)	15	0.02	4.24	2.04	$<10^{-5}$

Table.S6: Table summarizing the analytical model result for estimating the overall statistical significance of observing EE genes correlating significantly and positively/negatively with HyperZ/HypoZ across at least 6 of the 10 cancer types. Columns label the observed numbers of EE genes (nEE) correlating positively or negatively with either HyperZ or HypoZ across at least 6 cancer types, the null probability of observing a random gene (i.e. one of the ~20,000 genes) correlating significantly and consistently across at least 6 of the 10 cancer types (P, k≥6), the number of EE genes expected under the Binomial test (there were 212 EE genes), the standard deviation of the Binomial count (SD) and the corresponding Binomial test P-value. We can see that for a random set of 212 genes, that the expected numbers of genes correlating significantly and consistently with HyperZ/HypoZ across at least 6 of the 10 cancer types is significantly less than expected by random chance.

DNAm	BRCA	BLCA	COAD	HNSC	KIRC	LIHC	LSCC	LUAD	THCA	UCEC
paired normals	75	19	38	45	160	47	40	29	53	33
unpaired normals	6	0	0	0	0	0	1	3	0	1
cancer	652	201	272	405	299	176	275	399	489	374

RNA-seq	BRCA	BLCA	COAD	HNSC	KIRC	LIHC	LSCC	LUAD	THCA	UCEC
paired normals	96	17	26	41	71	50	45	57	56	10
unpaired normals	1	0	15	1	1	0	0	1	0	0
cancer	1008	323	270	475	515	349	473	471	495	364

Table.S7: Table summarizing the number of paired and unpaired normals and cancer samples for each of the 10 TCGA cancer types considered in this manuscript. We note that unpaired normal samples may still represent tissue adjacent to cancer, but don't have a corresponding cancer sample (RNA or DNA) available. Only for colon (COAD) there were sufficient numbers of unpaired normals, with 12 of the 15 RNA-Seq samples not having any matched cancer sample (be it RNA-Seq or DNAm).

Supplementary References:

1. Shen, H. & Laird, P.W. Interplay between the cancer genome and epigenome. *Cell* **153**, 38-55 (2013).
2. Plass, C. et al. Mutations in regulators of the epigenome and their connections to global chromatin patterns in cancer. *Nature reviews. Genetics* **14**, 765-780 (2013).
3. Koboldt, D.C. et al. Comprehensive molecular portraits of human breast tumours. *Nature* **490**, 61-70 (2012).
4. Cancer Genome Atlas Research, N. Comprehensive molecular characterization of urothelial bladder carcinoma. *Nature* **507**, 315-322 (2014).
5. Cancer Genome Atlas, N. Comprehensive molecular characterization of human colon and rectal cancer. *Nature* **487**, 330-337 (2012).
6. Cancer Genome Atlas, N. Comprehensive genomic characterization of head and neck squamous cell carcinomas. *Nature* **517**, 576-582 (2015).
7. Cancer Genome Atlas Research, N. Comprehensive molecular characterization of clear cell renal cell carcinoma. *Nature* **499**, 43-49 (2013).
8. Kechavarzi, B. & Janga, S.C. Dissecting the expression landscape of RNA-binding proteins in human cancers. *Genome biology* **15**, R14 (2014).
9. Cancer Genome Atlas Research, N. Comprehensive molecular profiling of lung adenocarcinoma. *Nature* **511**, 543-550 (2014).

10. Cancer Genome Atlas Research, N. Comprehensive genomic characterization of squamous cell lung cancers. *Nature* **489**, 519-525 (2012).
11. Cancer Genome Atlas Research, N. Integrated genomic characterization of papillary thyroid carcinoma. *Cell* **159**, 676-690 (2014).
12. Cancer Genome Atlas Research, N. et al. Integrated genomic characterization of endometrial carcinoma. *Nature* **497**, 67-73 (2013).
13. Teschendorff, A.E. et al. An epigenetic signature in peripheral blood predicts active ovarian cancer. *PloS one* **4**, e8274 (2009).
14. Teschendorff, A.E., Zhuang, J. & Widschwendter, M. Independent surrogate variable analysis to deconvolve confounding factors in large-scale microarray profiling studies. *Bioinformatics* **27**, 1496-1505 (2011).
15. Sandoval, J. et al. Validation of a DNA methylation microarray for 450,000 CpG sites in the human genome. *Epigenetics : official journal of the DNA Methylation Society* **6**, 692-702 (2011).
16. Troyanskaya, O. et al. Missing value estimation methods for DNA microarrays. *Bioinformatics* **17**, 520-525 (2001).
17. Teschendorff, A.E. et al. A beta-mixture quantile normalization method for correcting probe design bias in Illumina Infinium 450 k DNA methylation data. *Bioinformatics* **29**, 189-196 (2013).
18. Smyth, G.K. Linear models and empirical bayes methods for assessing differential expression in microarray experiments. *Statistical applications in genetics and molecular biology* **3**, Article3 (2004).
19. Aryee, M.J. et al. Minfi: a flexible and comprehensive Bioconductor package for the analysis of Infinium DNA methylation microarrays. *Bioinformatics* **30**, 1363-1369 (2014).
20. Jaffe, A.E. et al. Bump hunting to identify differentially methylated regions in epigenetic epidemiology studies. *International journal of epidemiology* **41**, 200-209 (2012).
21. Opgen-Rhein, R. & Strimmer, K. From correlation to causation networks: a simple approximate learning algorithm and its application to high-dimensional plant gene expression data. *BMC systems biology* **1**, 37 (2007).
22. Parker, J.S. et al. Supervised risk predictor of breast cancer based on intrinsic subtypes. *Journal of clinical oncology : official journal of the American Society of Clinical Oncology* **27**, 1160-1167 (2009).
